# Metabolic compensation activates pro-survival mTORC1 signaling upon 3-phosphoglycerate dehydrogenase inhibition in osteosarcoma

**DOI:** 10.1016/j.celrep.2020.108678

**Published:** 2021-01-26

**Authors:** Richa Rathore, Katharine E. Caldwell, Charles Schutt, Caitlyn B. Brashears, Bethany C. Prudner, William R. Ehrhardt, Cheuk Hong Leung, Heather Lin, Najat C. Daw, Hannah C. Beird, Abigail Giles, Wei-Lien Wang, Alexander J. Lazar, John S.A. Chrisinger, J. Andrew Livingston, Brian A. Van Tine

**Affiliations:** 1 Department of Medicine, Division of Medical Oncology, Washington University in St. Louis, St. Louis, MO 63110, USA; 2 Department of Surgery, Division of Hepatobiliary Surgery, Washington University in St. Louis, St. Louis, MO 63110, USA; 3 Department of Biostatistics, University of Texas MD Anderson Cancer Center, Houston, TX 77030, USA; 4 Division of Pediatrics, University of Texas MD Anderson Cancer Center, Houston, TX 77030, USA; 5 Department of Genomic Medicine, University of Texas MD Anderson Cancer Center, Houston, TX 77030, USA; 6 Department of Pathology, University of Texas MD Anderson Cancer Center, Houston, TX 77030, USA; 7 Department of Pathology and Immunology, Washington University in St. Louis, St. Louis, MO 63110, USA; 8 Department of Sarcoma Medical Oncology, University of Texas MD Anderson Cancer Center, Houston, TX 77030, USA; 9 Siteman Cancer Center, St. Louis, MO 63110, USA; 10 Lead contact

## Abstract

Osteosarcoma is the most common pediatric and adult primary malignant bone cancer. Curative regimens target the folate pathway, downstream of serine metabolism, with high-dose methotrexate. Here, the rate-limiting enzyme in the biosynthesis of serine from glucose, 3-phosphoglycerate dehydrogenase (PHGDH), is examined, and an inverse correlation between PHGDH expression and relapse-free and overall survival in osteosarcoma patients is found. PHGDH inhibition in osteosarcoma cell lines attenuated cellular proliferation without causing cell death, prompting a robust metabolic analysis to characterize pro-survival compensation. Using metabolomic and lipidomic profiling, cellular response to PHGDH inhibition is identified as accumulation of unsaturated lipids, branched chain amino acids, and methionine cycle intermediates, leading to activation of pro-survival mammalian target of rapamycin complex 1 (mTORC1) signaling. Increased mTORC1 activation sensitizes cells to mTORC1 pathway inhibition, resulting in significant, synergistic cell death *in vitro* and *in vivo*. Identifying a therapeutic combination for PHGDH-high cancers offers preclinical justification for a dual metabolism-based combination therapy for osteosarcoma.

## In Brief

Rathore et al. identify 3-phosphoglycerate dehydrogenase (PHGDH) overexpression as a marker of poor prognosis in osteosarcoma. PHGDH inhibition causes decreased proliferation, inhibited TCA cycle, and accumulation of metabolites that drive the activation of mTORC1. Non-rapalog mTORC1 inhibition combined with PHGDH inhibition demonstrates a possible synergistic dual metabolic therapy for osteosarcoma.

## Graphical Abstract

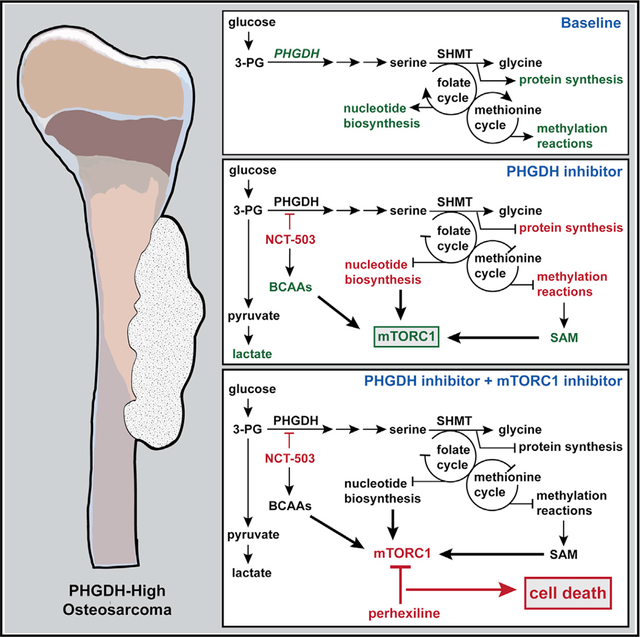

## INTRODUCTION

Osteosarcoma is the most common primary malignant bone tumor in both children and adults, accounting for approximately 4% of all childhood cancers and 56% of pediatric malignant bone tumors (Geller and Gorlick, 2010). Current curative therapy for osteosarcoma is neoadjuvant chemotherapy, consisting of cisplatin, doxorubicin, and high-dose methotrexate; resection; and additional cycles of chemotherapy (Heare et al., 2009). High-dose methotrexate has been a key component of standard-of-care treatment for osteosarcoma since the 1970s, based on small cohort clinical trials that measured levels of tumor necrosis at the time of surgery (Grem et al., 1988; Rosen et al., 1974, 1979). Although these trials showed that most patients demonstrated little viable tumor tissue with methotrexate treatment, it was noted that high levels of toxicity were associated with this treatment (Li et al., 2019; Perez et al., 1978; Rosen et al., 1974, 1979). The doses required for histologic response were as high as 8–20 g/m^2^, which can result in kidney injury or liver toxicity, especially in adult patients (Howard et al., 2016; Rosen et al., 1974; Wippel et al., 2019). Therefore, identifying less-toxic therapies for osteosarcoma is a goal of the field (Shaikh et al., 2016).

A variety of molecular targeted therapies have been investigated for osteosarcoma. Recently, clinical trials have focused on tyrosine kinase inhibitors (Davis et al., 2019; Italiano et al., 2020). The mammalian target of rapamycin (mTOR) has also been a target of interest in osteosarcoma; however, single-agent studies of mTOR inhibitors have failed in osteosarcoma trials (Ding et al., 2016). Combinations of mTOR inhibitors with inhibitors of other pathways, including a trial combining the tyrosine kinase inhibitor sorafenib with mTOR inhibition (Grignani et al., 2015), have also been explored, suggesting that molecular targets based on tumor-specific biomarkers are an active avenue for the development of novel therapies. However, no agents have been added to the front-line curative therapy since the addition of high-dose methotrexate.

Methotrexate inhibits dihydrofolate reductase (DHFR), a component of the folate cycle. The folate cycle catalyzes the production of purines and pyrimidines within the cell. Methotrexate-mediated inhibition of DHFR decreases tetrahydrofolate levels, resulting in decreased methylation, impaired DNA synthesis, and diminished nucleotide synthesis (Tian and Cronstein, 2007). These effects have made the folate cycle, and more broadly, one-carbon metabolism, a key player in the evolution of cancer research (Ducker and Rabinowitz, 2017).

The enzyme serine hydroxymethyl transferase (SHMT) catalyzes the conversion of the amino acid serine and tetrahydrofolate (THF) to glycine and 5,10-methylene-THF, thus transferring a one-carbon unit into the folate cycle (García-Cañaveras et al., 2020). Proliferating cells can obtain serine exogenously or through *de novo* serine biosynthesis from glucose. The upregulation of the rate-limiting enzyme of serine biosynthesis, 3-phosphoglycerate dehydrogenase (PHGDH), has been identified in a large subset of cancers, including melanoma (Mullarky et al., 2011), Ewing’s sarcoma (Issaq et al., 2020), breast (Locasale et al., 2011; Possemato et al., 2011), colorectal (Jia et al., 2016), pancreatic (Song et al., 2018), and non-small cell lung cancers (Zhu et al., 2016). PHGDH converts the glycolytic intermediate 3-phosphoglycerate (3PG) into 3-phosphohydroxypyruvate (3PP), producing NADH from NAD^+^ as a reaction byproduct (Fan et al., 2015). Increased PHGDH levels and subsequent increases in *de novo* serine biosynthesis result in the upregulation of the cellular events fueled by the folate and methionine cycles, including increased DNA synthesis, increased biomass production, and increased methylation processes, and can also contribute to tumor stem cell differentiation (Baksh et al., 2020; Possemato et al., 2011).

Given the importance of DHFR inhibition, PHGDH inhibition was explored as a possible replacement for methotrexate. NCT-503 was identified in a screen of PHGDH inhibitors to have inhibitory capacity *in vitro* and *in vivo* (Pacold et al., 2016). Several other small-molecule PHGDH inhibitors have been identified to have similar cytostatic effects *in vitro* (Mullarky et al., 2016; Wang et al., 2017). Investigation of broader metabolic changes from PHGDH inhibition have highlighted glycolytic flux of glucose, as well as increased total intracellular serine (Pacold et al., 2016). Notably, PHGDH inhibition in PHGDH-high cell lines has primarily shown a decrease in proliferation, with cell death seen at high concentrations that are likely not clinically translatable (Pacold et al., 2016).

This study demonstrates that high levels of PHGDH correlated with poor overall relapse-free and poor overall survival outcomes in patients with osteosarcoma. PHGDH inhibition has a cytostatic effect, consistent with previous publications in other PHGDH-high cancers (Mullarky et al., 2016; Wang et al., 2017; Wei et al., 2019). Given the metabolic systems associated with serine biology, metabolomics, lipidomics, and gene-expression analyses were conducted. These data showed that cells treated with the PHGDH inhibitor NCT-503 accumulated a series of metabolites, including unsaturated fatty acids and branched chain amino acids, which resulted in pro-survival activation of the mTOR complex 1 (mTORC1) signaling network. Cells treated with a combination of NCT-503 and perhexiline, a non-rapalog inhibitor of the mTORC1 pathway (Balgi et al., 2009), exhibited significant cell death. The mechanism of perhexiline in this system was explored, with the accumulation of branched chain amino acids and methionine cycle intermediates suggesting involvement of the SAMTOR-GATOR1-KICSTOR pathway (Gu et al., 2017; Wolfson et al., 2017). Taken together, these data suggest that activation of the mTORC1 signaling pathway by PHGDH inhibition can specifically sensitize cells to treatment with non-rapalog mTORC1 inhibitors. These findings offer the preclinical justification for a dual metabolic therapy for osteosarcoma.

## RESULTS

### Folate cycle inhibition results in cytostasis *in vitro*

**Figure 1.**
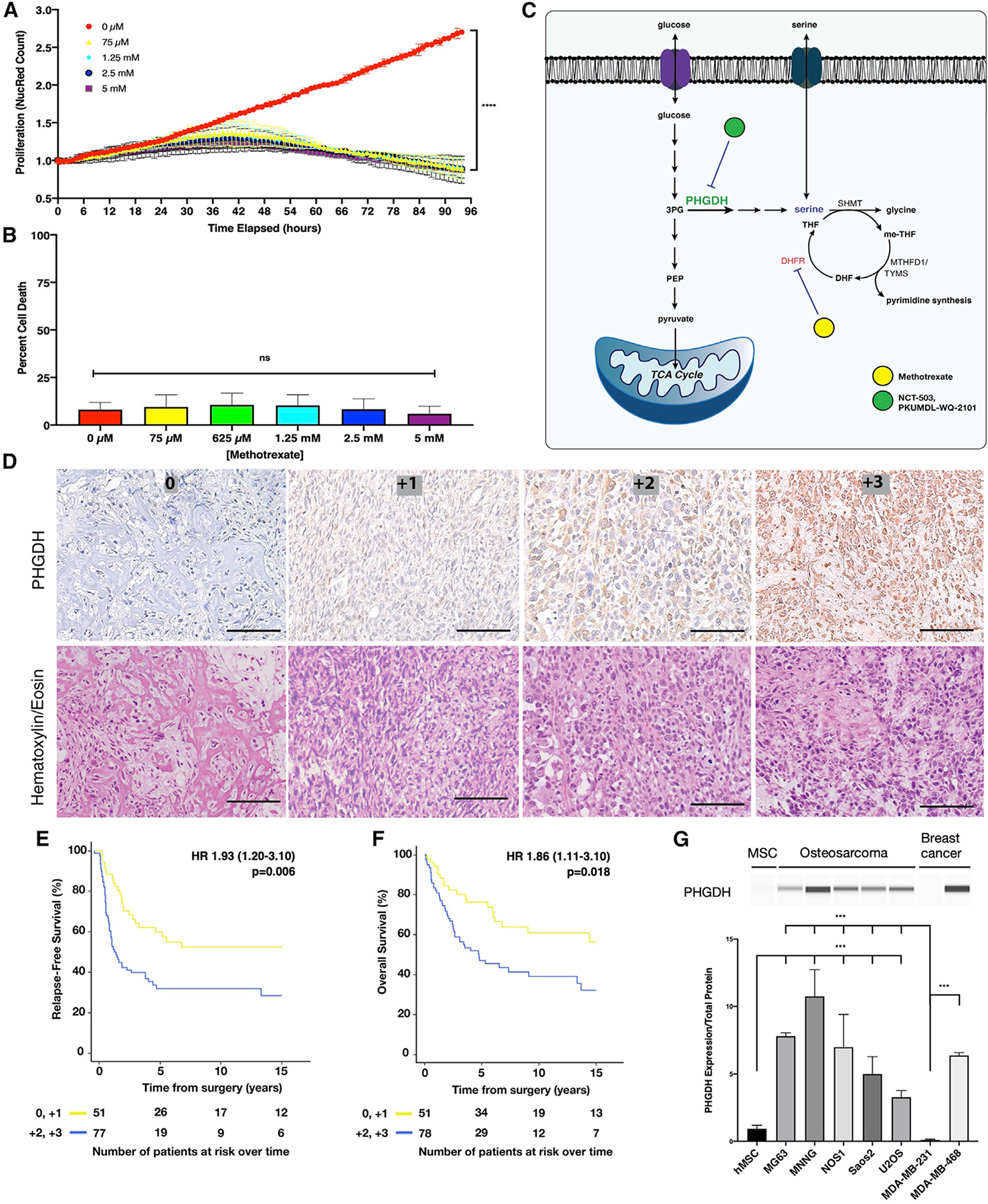
Osteosarcomas exhibit cytostasis with folate cycle inhibition and demonstrate correlation between upstream PHGDH expression and poor patient prognosis (A) Nuclear red count in NOS1 cell lines treated with increasing doses of methotrexate for up to 96 h, normalized to starting nuclear red count. (B) Percentage of cell death at 72 h in the NOS1 cell line treated with increasing doses of methotrexate. (C) Schematic of metabolic pathways linking folate metabolism to *de novo* serine synthesis pathway. (D) Representative immunohistochemistry for PHGDH of a tumor microarray containing 392 tumor samples from 260 osteosarcoma patients, with hematoxylin and eosin cross-staining. Scale bars represent 100 μm. (E) Relapse-free survival for osteosarcoma patients with medium to high levels of PHGDH (blue) compared with none to low levels of PHGDH (yellow). (F) Overall survival for patients with osteosarcoma and medium to high levels of PHGDH (blue) compared with those with none to low levels of PHGDH (yellow). (G) Protein expression of PHGDH in precursor mesenchymal stem cell; osteosarcoma cell lines MG63, MNNG, NOS1, Saos2, and U2OS; and breast cancer cell lines MDA-MB-231 and MDA-MB-468. Cell lysates were analyzed using the Wes automated capillary blotting system, normalized to total protein levels, and a representative band image was used. 3PG, 3-phosphoglycerate; PEP, phosphoenolpyruvate; PHGDH, 3-phosphoglycerate dehydrogenase; SHMT, serine hydroxymethyl transferase; me-THF, methylene tetrahydrofolate; MTHFD1, methylene tetrahydrofolate dehydrogenase; TYMS, thymidylate synthetase; DHF, dihydrofolate; DHFR, dihydrofolate reductase; THF, tetrahydrofolate. Bars represent means of values; error bars represent SEM. All assays were conducted with n = 3 replicates. *p < 0.05, **p < 0.01, ***p < 0.005, ****p < 0.001.

To test the effect of methotrexate, the osteosarcoma cell line NOS1 was cultured in the presence of increasing doses of methotrexate for up to 96 h, and cellular proliferation rate was measured using the IncuCyte ZOOM live cell analysis system (Figure 1A). Although proliferation was inhibited with high doses of methotrexate (greater than 75 μM), a decrease in cell count at high doses was not observed, suggesting cytostasis and not cell death. To address this, cell death was measured using YOYO-1 iodide fluorescence (Figure 1B). Methotrexate caused no significant cell death up to concentrations as high as 5 mM at 48 h. These findings demonstrate that exceedingly high doses of methotrexate are required to show an anti-proliferative effect *in vitro*.

### PHGDH expression correlates with poor patient prognosis in osteosarcoma

Given the lack of cell death induced by methotrexate, upregulation of the serine biosynthetic pathway was investigated (Figure 1C). Expression of PHGDH, the rate-limiting enzyme in serine biosynthesis, was characterized in a triple-blind fashion using immunohistochemistry on a tumor microarray of 392 tumor samples from 260 patients with osteosarcoma (Figure 1D; patient characteristics are presented in Table S1). A blinded, independent pathologist scored tumors for staining intensity by standard pathology grading, as 0 (no PHGDH), +1 (low PHGDH), +2 (medium PHGDH), or +3 (high PHGDH). These blinded scores were then correlated with survival data for the tumor microarray by a blinded, independent biostatistician.

Of the tumors assayed, 53% (208/392) were found to have medium to high expression of PHGDH. Furthermore, medium to high levels of PHGDH were associated with decreased relapse-free survival (median relapse-free survival [RFS] 1.25 years [low] versus >15 years [not reached; medium/high]) (hazard ratio [HR] 1.93 [range 1.20–3.10], p = 0.006; Figure 1E) and decreased overall survival (OS; median OS 4.6 years [low] versus >15 years [not reached; medium/high]) (HR 1.86 [range 1.11–3.10], p = 0.018; Figure 1F) in resection samples from patients with osteosarcoma and localized disease who received neoadjuvant chemotherapy. This demonstrated that high PHGDH expression was correlated with osteosarcomas with poorer outcomes. Using a second available dataset from patients with osteosarcoma, which were selected from cases with poor outcomes because of relapsed or metastatic disease, high PHGDH expression by RNA sequencing (RNA-seq) was also correlated with poor disease-free survival (DFS; p = 0.031; Figures S1A and patient characteristics presented in S1B). Given the selection bias in the dataset, the correlation between high PHGDH expression and poor OS was consistent but not significant (Figure S1C).

Osteosarcoma cell lines (MG63, MNNG, NOS1, Saos2, and U2OS), human mesenchymal stem cells (the progenitor cell to bone-producing cells), and two breast cancer cell lines (MDA-MB-231 and MDA-MB-468) with known PHGDH status (null and high, respectively) were selected for further *in vitro* analysis of PHGDH biology (Pacold et al., 2016). All osteosarcoma cell lines were found to have PHGDH levels that were significantly higher than those of mesenchymal cells or the known-negative breast-cancer cell line (Figure 1G).

### PHGDH inhibition causes attenuation of cellular proliferation

**Figure 2.**
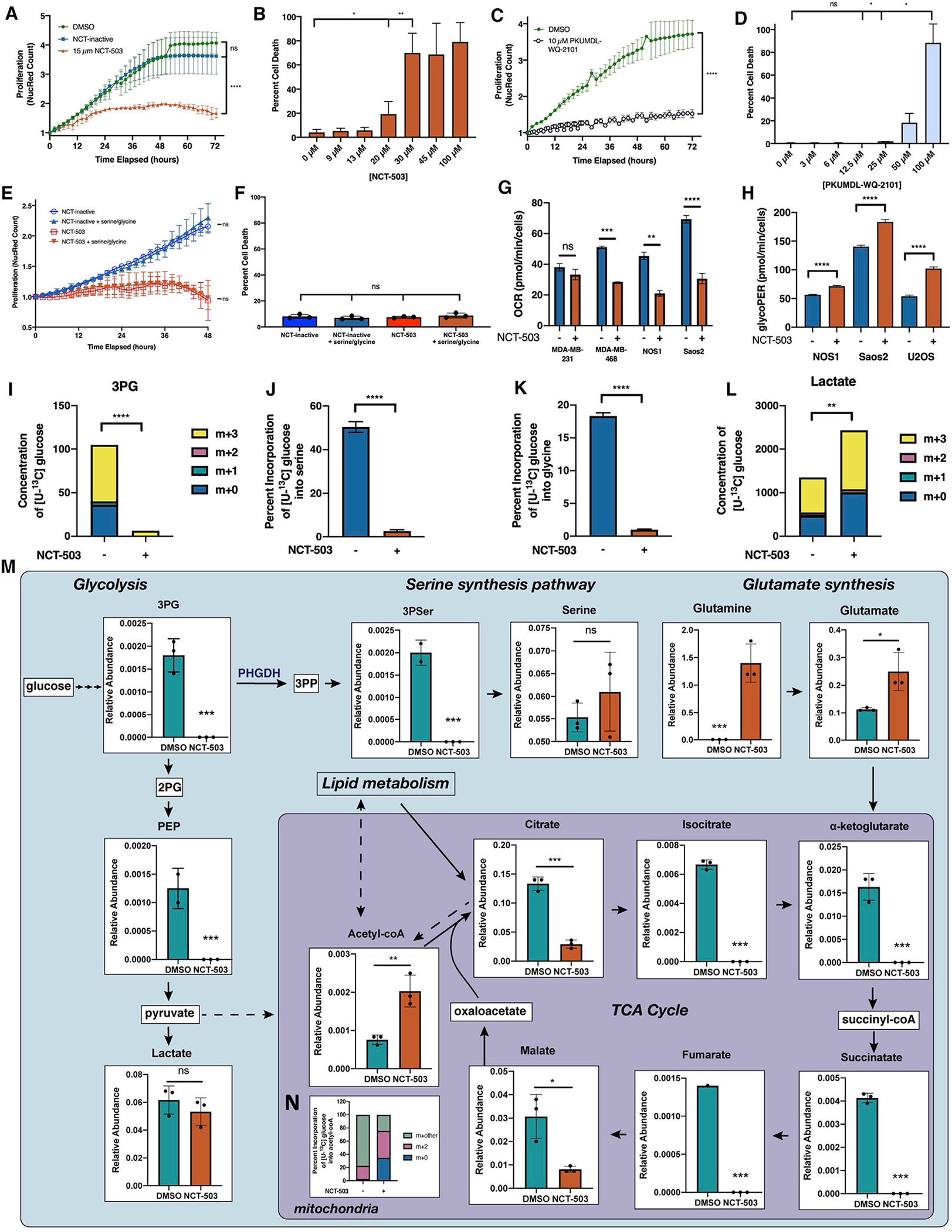
PHGDH inhibition causes attenuation of cellular proliferation and TCA cycle activity (A) Nuclear red count in NOS1 cells treated with DMSO (vehicle control), 10 μM NCT-503 inactive control, or 15 μM NCT-503. (B) Percentage of cell death at 72 h in NOS1 cells treated with increasing doses of NCT-503. (C) Nuclear red count in NOS1 cells treated with DMSO or 10 μM PKUMDL-WQ-2101. (D) Percentage of cell death at 72 h in NOS1 cells treated with increasing doses of PKUMDL-WQ-2101. (E and F) Nuclear red count (E) and percentage of cell death (F) in NOS1 cells cultured with media containing dialyzed fetal bovine serum (FBS) ± supplementation with 286 μM serine and glycine and treated with NCT-inactive or NCT-503. (G) Oxygen consumption rate (OCR) for MDA-MB-231, MDA-MB-468, NOS1, and Saos2 cells treated with NCT-inactive or NCT-503. (H) Glycolytic proton efflux rate (GlycoPER) for NOS1, Saos2, and U2OS cells treated with NCT-inactive or NCT-503. (I) Concentration of ^13^C and unlabeled C in 3PG in Saos2 cells treated with NCT-inactive or NCT-503. (J and K) Percentage of incorporation of [U-^13^C] labeled glucose into serine (J) and glycine in Saos2 cells treated with NCT-inactive or NCT-503 (K). (L) Concentration of ^13^C and unlabeled C in lactate in Saos2 cells treated with NCT-inactive or NCT-503. (M) Metabolite levels in NOS1 cells treated with DMSO or NCT-503 for 48 h. Dashed lines indicate potential sources of acetyl-coA. (N) Percentage of incorporation of [U-^13^C] labeled glucose into carbons of acetyl-coA in Saos2 cells treated with NCT-inactive or NCT-503. 3PP, 3-phosphohydroxypyruvate; 3PSer, 3-phophoserine. Bars represent means of values; error bars represent SEM. All assays were conducted with n = 3 replicates. *p < 0.05, **p < 0.01, ***p < 0.005, ****p < 0.001.

To test the effect of inhibiting PHGDH in osteosarcoma cell lines, NCT-503, a small-molecule inhibitor of PHGDH, or an NCT-503-inactive control (NCT-inactive) was used. PHGDH inhibition caused significant attenuation of cellular proliferation with 15 μM NCT-503 in NOS1 (Figure 2A), as well as Saos2 and U2OS (Figures S2A and S2B), but no cell death was observed until concentrations of 20 μM (Figure 2B). Greater than 50% cell death was not seen until concentrations of 30 μM or higher; at which point, cell death could not be directly attributed to PHGDH inhibition because of the potential for off-target effects. The inhibitory capacity of NCT-503 on PHGDH has been reported (Pacold et al., 2016) and was assessed in osteosarcoma cell lines using a PHGDH activity assay (Figure S2C). EC-50 values for NCT-503 were calculated for each cell line (Figure S2D), and 15 μM NCT-503 was used for *in vitro* experiments for the remainder of the study.

To recapitulate the proliferation phenotype with another small-molecule inhibitor of PHGDH, the effects of PKUMDL-WQ-2101 in this system were also assayed, showing that 10 μM of PKUMDL-WQ-2101 could slow cellular proliferation (Figure 2C), whereas concentrations of greater than 50 μM were required to see greater than 50% cell death (Figure 2D). Finally, to verify that the decrease in proliferation was due to the on-target inhibition of PHGDH, stable knockdown cell lines of PHGDH were generated using short hairpin RNA (shRNA). PHGDH knockdown decreased proliferation rates in U2OS but had no effect on proliferation in the reference PHGDH-negative breast cancer cell line, MDA-MB-231 (Figures S2E and S2F).

Previous reports of PHGDH inhibition have shown that PHGDH-high cells were more sensitive to NCT-503 in the absence of serine and glycine (Mullarky et al., 2016). NOS1 cells were therefore cultured in medium supplemented with dialyzed FBS, with and without serine and glycine. NCT-503 treatment decreased the proliferation rate of osteosarcoma, but this was not dependent on the presence or absence of serine and glycine (Figure 2E), and there was no increase in cell death at 48 h (Figure 2F). This suggests that osteosarcoma cell lines with high levels of PHGDH likely are not dependent on extracellular serine.

### Inhibition of serine synthesis blocks TCA cycle activity

To identify the pro-survival compensatory mechanisms associated with PHGDH inhibition, the mitochondrial respiration of cells treated with NCT-503 was explored. There was no change in oxygen consumption rate in PHGDH-negative breast cancer cell line MDA-MB-231 after PHGDH inhibition with NCT-503, whereas osteosarcoma (NOS1 and Saos2) and PHGDH-positive breast cancer (MDA-MB-468) cell lines showed significant decreases in oxygen consumption rates (Figure 2G). This identified broader effects on the cells than the direct obstruction of serine biosynthesis by PHGDH inhibition.

Given that inhibition of PHGDH would limit 3-phosphoglycerate (3PG) incorporation into *de novo* serine synthesis, the fate of glucose and 3PG in this system was explored. With PHGDH inhibition, the glycolytic rate in osteosarcoma cell lines was increased, demonstrating that, instead of entering *de novo* serine biosynthesis through 3PG, glucose was shunted through glycolytic intermediates (Figure 2H). [U-^13^C] glucose tracing in the Saos2 cell line demonstrated that total 3PG metabolite levels were decreased, with the 3PG present in the system fully labeled with ^13^C from extracellular glucose (Figure 2I). Glucose entry into serine biosynthesis was completely abrogated with PHGDH inhibition, with no ^13^C labeling in serine or glycine (Figures 2J and 2K), verifying the targeted effect of NCT-503. Levels of glycolytic intermediates downstream of the rate-limiting enzyme, phosphofructokinase, were decreased (Figure S2G), and glucose incorporation into lactate was increased (Figure 2L). Extracellular lactate levels were also independently measured and elevated (Figure S2H), further demonstrating increased glycolysis in cells treated with NCT-503.

To characterize the metabolic compensation occurring with PHGDH inhibition, metabolomics analyses were conducted for NOS1 with 48 h of NCT-503 treatment (full dataset presented in Table S2). At the time of measurement, levels of 3-phosphoserine (3PSer), the stable product of 3-phosphopyruvate, were decreased, demonstrating on-target inhibition of PHGDH by NCT-503 (Figure 2M). Levels of 3PG were decreased with NCT-503 treatment and glycolytic metabolites were depleted, resulting in increased lactate levels. This confirmed the glucose tracing results for glycolytic response. Metabolites in the tricarboxylic acid (TCA) cycle, including citrate, isocitrate, α-ketoglutarate (αKG), succinatate, fumarate, and malate, were also significantly decreased with PHGDH inhibition (Figures 2M and S2I). Given that αKG can be synthesized from glutamate through phosphoserine aminotransferase 1 (PSAT1), an enzyme in *de novo* serine biosynthesis, any αKG present after PHGDH inhibition was likely from sources other than glucose (Figure S2J). An accumulation of non-glucose-derived acetyl-coenzyme A (coA) was also identified (Figure 2N, potential sources of acetyl-coA indicated by dashed lines). Taken together, these data confirmed that NCT-503 treatment inhibited both *de novo* serine biosynthesis and the TCA cycle, correlating with the lack of mitochondrial respiration and increase in glycolysis in PHGDH-expressing cell lines as seen by Seahorse (Figures 2G and 2H).

### PHGDH inhibition causes accumulation of intracellular unsaturated fatty acids

Citrate can be reversibly converted into acetyl-coA to direct TCA cycle metabolites away from the mitochondria for fatty acid synthesis or for gene expression through histone acetylation (Shi and Tu, 2015). The decrease in citrate and accumulation of acetyl-coA showed that glucose was likely not entering the TCA cycle for mitochondrial respiration, and the accumulation of glutamine and glutamate suggested that glutamine was also not being used as an oxidative fuel source (Figure 2M). Treatment with BPTES showed no cytotoxic effect on osteosarcoma cells (Figure S3A), and levels of asparagine and aspartate were significantly decreased (Figures S3B and S3C), suggesting that, at baseline, these cells were not dependent on glutamine metabolism. Seahorse analyses were, therefore, performed to identify how the preferences of mitochondria for three fuel sources (glucose, glutamine, and fatty acids) changed with PHGDH inhibition.

**Figure 3.**
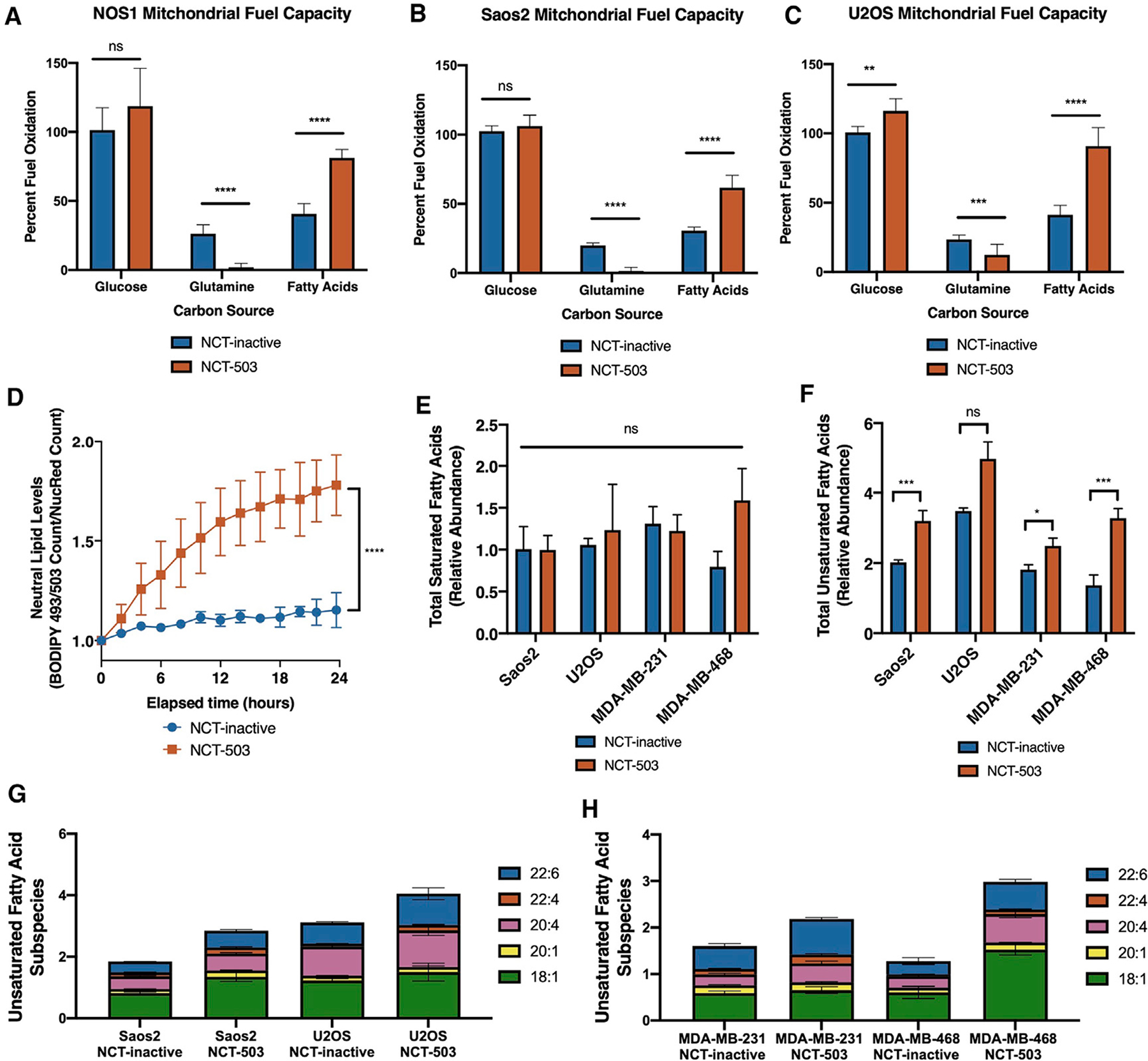
PHGDH inhibition causes accumulation of intracellular unsaturated fatty acids (A–C) Percentage of fuel oxidation of mitochondrial capacity to use fuel sources glucose, glutamine, and fatty acids, after treatment with 48 h of NCT-inactive or NCT-503 in NOS1 (A), Saos2 (B), and U2OS (C) cells. Bars represent means of values; error bars represent SEM n = 6 replicates. (D) Counts of green BODIPY 493/503 staining in NOS1 cells treated with NCT-inactive or NCT-503. (E and F) Relative abundance by peak area ratio of total saturated fatty acids (E) and total unsaturated fatty acids measured by liquid chromatography-mass spectrometry (LC-MS) (F) in Saos2, U2OS, MDA-MB-231, and MDA-MB-468 cells treated with NCT-inactive or NCT-503. (G and H) Relative abundance by peak area ratio of five most abundant unsaturated fatty acids in osteosarcoma cell lines (G) and breast cancer cell lines (H) (statistics presented in Figures S2C and S2D). Bars represent means of values; error bars represent SEM. All assays were conducted with n = 3 replicates unless otherwise specified. *p < 0.05, **p < 0.01, ***p < 0.005, ****p < 0.001.

In three osteosarcoma cell lines, treatment with NCT-503 resulted in increased capacity to use fatty acids (Figures 3A–3C), demonstrating that, when treated with NCT-503, osteosarcoma cells had an increased relative capacity to oxidize fatty acids when glucose or glutamine are absent. [U-^13^C] palmitate tracing identified increased ^13^C incorporation into *O*-acetylcarnitine and *N*-acetylaspartic acid, suggesting that a limited amount of palmitate was used for acetyl-coA synthesis, thereby supplementing lack of glucose incorporation into acetyl-coA (Figures 2M, S3D, and S3E). However, given the lack of TCA cycle respiration, mitochondrial usage of fatty acids for oxidative phosphorylation was unlikely. Therefore, BODIPY 493/503 fluorescence levels were measured with NCT-503 treatment over time, demonstrating that neutral fatty acids were accumulating intracellularly (Figure 3D).

To characterize the source of these lipids, levels of SREBP-1, a transcription factor that regulates triglyceride synthesis, were measured (Eberlé et al., 2004). PHGDH inhibition did not change the protein expression of SREBP-1 (Figure S3F), suggesting the accumulation of lipids was not due to increased synthesis. Cells were then cultured in medium containing normal or lipid-depleted FBS and treated with NCT-503. Depletion of lipids from the culture media did not significantly change cell death with NCT-503 treatment (Figure S3G). To identify the lipids that were accumulating, unbiased lipidomics analyses for free fatty acids (unsaturated and saturated), phospholipids, and cholesterol esters were conducted in cells treated with NCT-503 or NCT-inactive control (full dataset presented in Table S3). There was no change in phospholipids, cholesterol esters, or saturated fatty acids (Figure 3E; Table S3), but an increase in unsaturated fatty acids with NCT-503 treatment (Figure 3F). The lipidomic analysis also indicated changes in unsaturated fatty acid subspecies, from which the five most abundant demonstrated significant increases with NCT-503 treatment (Figures 3G, 3H, and statistics presented in S3H and S3I). Taken together, these findings confirmed that PHGDH inhibition by NCT-503 caused a decrease in mitochondrial respiration and an accumulation of metabolites and fatty acids that feed into the mitochondria.

### Inhibition of serine synthesis and subsequent accumulation of branched chain amino acids leads to mTORC1 activation

**Figure 4.**
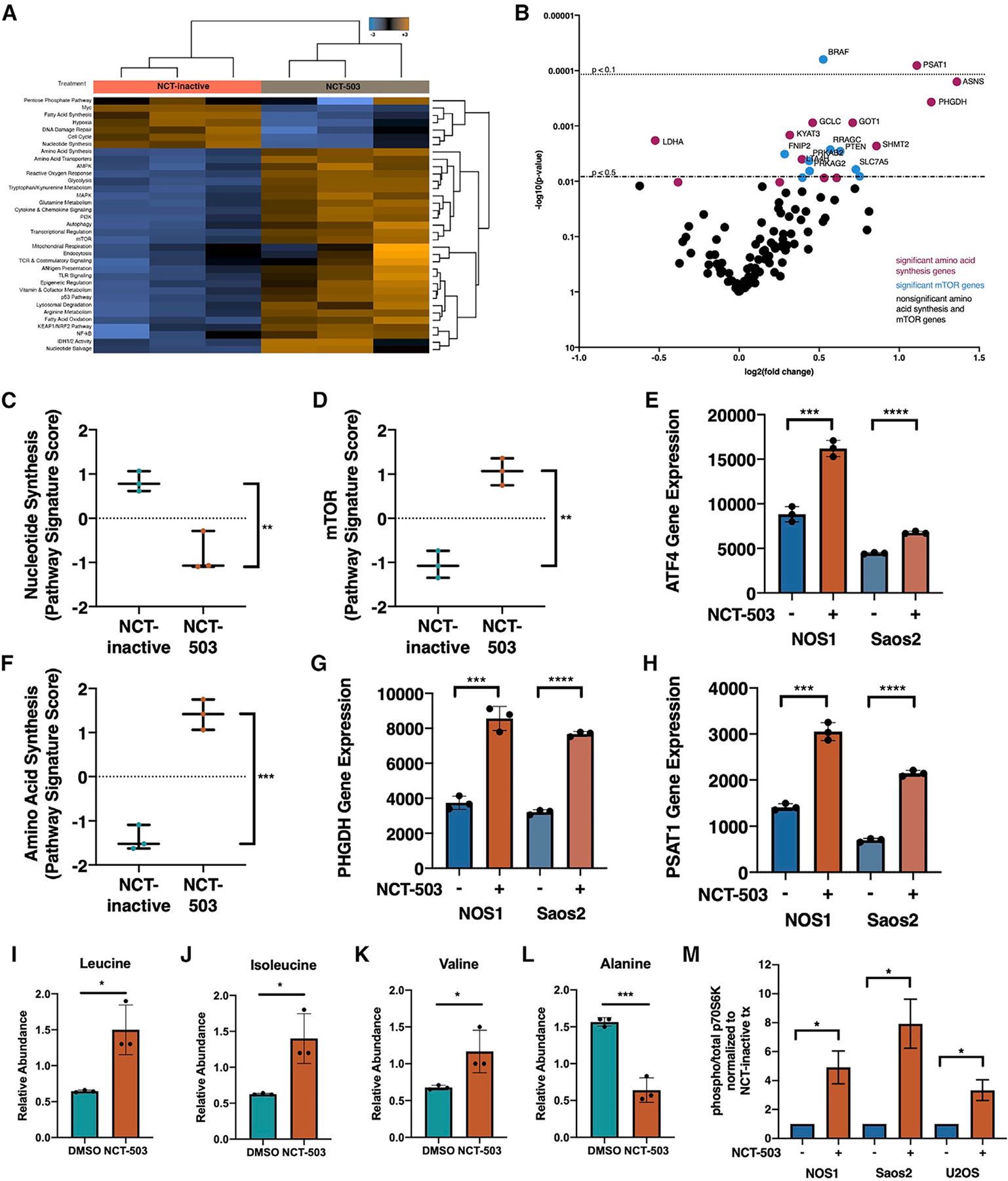
Inhibition of serine synthesis and subsequent accumulation of branched chain amino acids leads to mTORC1 activation (A) Heatmap of pathway scores for pathways characterized by NanoString Metabolism Gene Panel in NOS1 cells treated with NCT-inactive or NCT-503. Shades of orange indicate upregulation; shades of blue indicate downregulation. (B) Gene set analysis plot for significant and non-significant (black) genes in amino acid synthesis pathway (purple) and mTOR pathway (blue). (C) Pathway signature score for nucleotide synthesis pathway and (D) mTOR pathway in NOS1 cells treated with NCT-inactive or NCT-503, relative to NCT-inactive. (E) Linear normalized gene counts for ATF4 in NOS1 and Saos2 cells treated with NCT-inactive or NCT-503. (F) Pathway signature score for amino acid synthesis pathway in NOS1 cells treated with NCT-inactive or NCT-503, relative to NCT-inactive. (G and H) Linear normalized gene counts for PHGDH (G) and PSAT1 (H) in NOS1 and Saos2 cells treated with NCT-inactive or NCT-503. (I–L) Levels of amino acids in NOS1 cells treated with DMSO or NCT-503 for 48 h, including leucine (I), isoleucine (J), valine (K), and alanine (L). (M) Ratio of protein expression of phosphorylated (Thr389) p70S6K to total p70S6K in NOS1, Saos2, and U2OS cell lines in presence of NCT-inactive or NCT-503. Cell lysates were analyzed using Wes automated capillary blotting system, normalized to total protein levels, and a representative band image was used. Bars represent means of values; error bars represent SEM. All assays were conducted with n = 3 replicates. *p < 0.05, **p < 0.01, ***p < 0.005, ****p < 0.001.

To determine additional metabolic compensation that cells undergo with NCT-503 treatment, a metabolic gene expression analysis was conducted using the NanoString metabolism gene-panel analysis (full dataset presented in Table S4). Genes were assigned to pathways through the nSolver Advanced Analysis system, and scores were generated that showed upregulation or downregulation of metabolic pathways as a whole (Figure 4A). Several genes related to cellular nutrient and fatty acid sensing, including amino acid synthesis genes and mTOR pathway genes, were significantly upregulated, suggesting a role of these pathways in the pro-survival transcriptional adaptations of the cell (Figure 4B). Plotting the pathway scores calculated for nucleotide synthesis, the result of carbon input into the folate cycle, showed that genes related to nucleotide synthesis were largely downregulated (Figure 4C). This was corroborated by significantly decreased metabolite levels in the purine metabolism (p = 0.0003) and pyrimidine metabolism (p < 0.0001) pathways (Figure S4A). Importantly, genes related to the mTOR pathway, which is directly involved in nucleotide sensing in the cell, were significantly upregulated (Figure 4D).

mTORC1 increases purine and pyrimidine biosynthesis by activating ATF4 (Ben-Sahra et al., 2016). NanoString analysis showed that *ATF4* expression was significantly upregulated with NCT-503 treatment (Figure 4E). *ATF4* increases transcription of serine synthesis genes (Zhao et al., 2016). The metabolomics pathway scores for amino acid synthesis were also increased with NCT-503 treatment (Figure 4F). PHGDH inhibition resulted in increased gene expression of serine synthetic pathway enzymes *PHGDH* (Figure 4G) and *PSAT1* (Figure 4H).

In addition to sensing nucleotide levels, mTORC1 signaling is highly responsive to changes in amino acid levels (Dyachok et al., 2016). Gene expression profiling showed an increase in serine biosynthetic enzymes, indicating a targeted response to PHGDH inhibition. PHGDH inhibition also resulted in increased intracellular concentrations of branched-chain amino acids (BCAAs) leucine, isoleucine, and valine (Figures 4I–4K) and increased expression of BCAA transporters *SLC7A5* and *SLC3A2* (Figures S4B and S4C). Importantly, not all amino acids levels were increased in the cell; for example, alanine, an amino acid that can be synthesized from pyruvate, decreased with NCT-503 treatment (Figure 4L). Confirming that these regulatory and nutrient stimuli were resulting in mTORC1 activation, levels of threonine 389 (Thr389) phosphorylation on p70 S6 kinase were elevated with NCT-503 treatment (Figure 4M). These data demonstrate that cellular sensing of decreased nucleotide synthesis and changes in amino acid levels because of PHGDH inhibition drives an activation of the mTOR signaling pathway.

### Perhexiline, but not rapamycin, causes cell death in osteosarcoma and other PHGDH-high cell lines when combined with PHGDH inhibition

**Figure 5.**
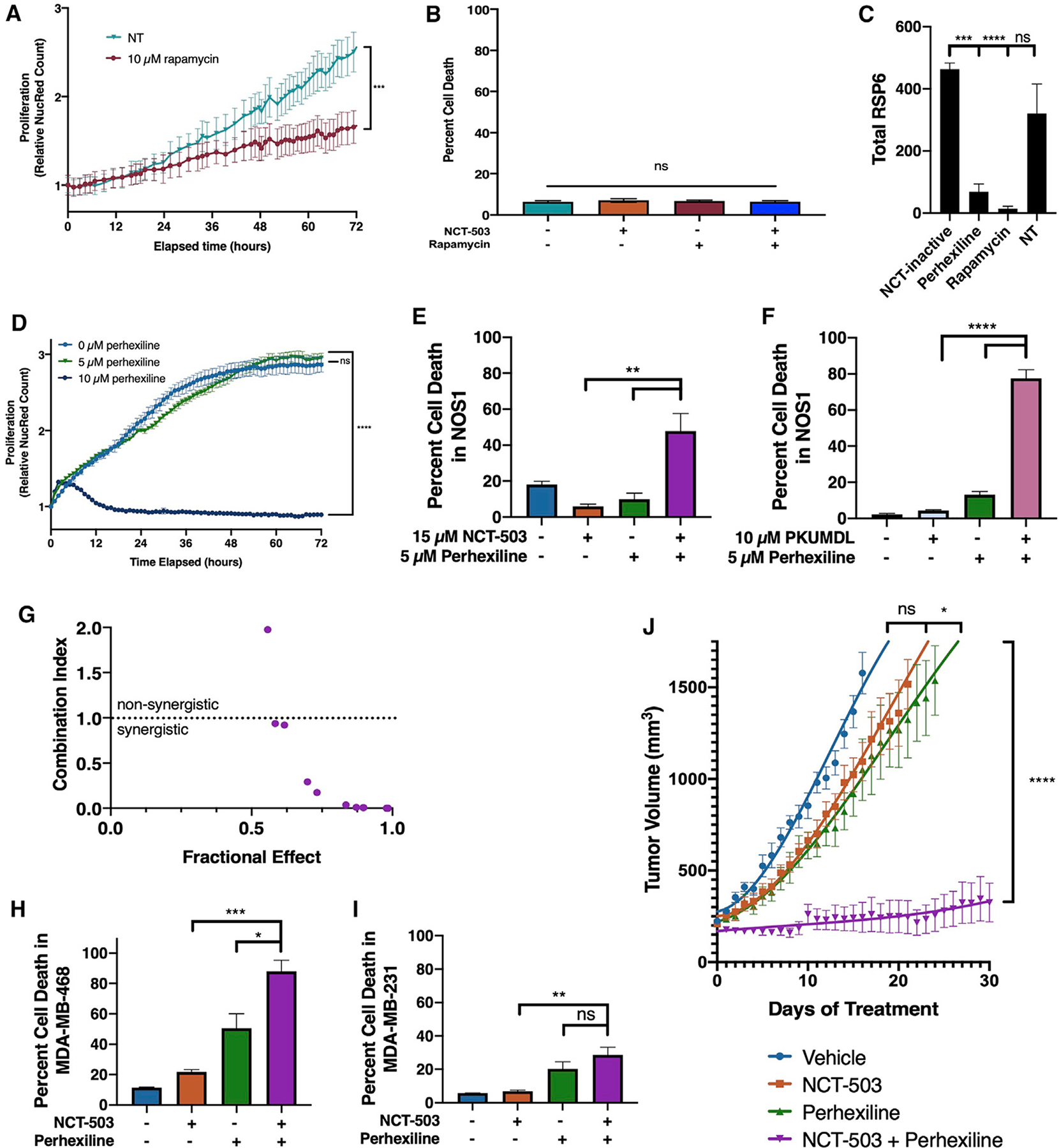
Perhexiline, but not rapamycin, causes cell death in osteosarcoma and other PHGDH-high cell lines when combined with PHGDH inhibition (A) Nuclear red count in NOS1 cells treated with DMSO (vehicle control) or 10 μM rapamycin. (B) Percentage of cell death at 72 h in NOS1 cells treated with DMSO (vehicle control), NCT-503, rapamycin, or a combination of NCT-503 and rapamycin. (C) Total protein levels of RSP6 in NOS1 cells treated with NCT-inactive, perhexiline, rapamycin, or a no treatment serum control. (D) Nuclear red count in NOS1 cells treated with DMSO (vehicle control), 5 μM perhexiline, or 10 μM perhexiline. (E) Percentage of cell death at 72 h in NOS1 cells treated with NCT-inactive, NCT-503, 5 μM perhexiline, or a combination of NCT-503 and 5 μM perhexiline. (F) Percentage of cell death at 72 h in NOS1 cells treated with 10 μM PKUMDL-WQ-2101, 5 μM perhexiline, or a combination of PKUMDL-WQ-2101 and 5 μM perhexiline. (G) Plot of combination index (CI) against fractional effect for the interaction of increasing doses of NCT-503 combined with increasing doses of perhexiline in NOS1 cells. CI > 1.0, antagonistic; 1.1 < CI < 0.9, additive; CI < 0.9, synergistic. (H and I) Percentage of cell death at 72 h in MDA-MB-468 (H) and MDA-MB-231 (I) cells treated with NCT-inactive, NCT-503, perhexiline, or a combination of NCT-503 and perhexiline. (J) Tumor volume of U2OS xenografts under various conditions: vehicle (n = 10), NCT-503 (n = 10), perhexiline (n = 10), and NCT-503 combined with perhexiline (n = 10). Bars represent means of values; error bars represent SEM. All assays were conducted with n = 3 replicates unless otherwise specified. *p < 0.05, **p < 0.01, ***p < 0.005, ****p < 0.001.

Accumulation of BCAAs has been shown to activate mTOR signaling through Sestrin-mediated GATOR2 activity (Kim et al., 2015; Nicklin et al., 2009; Persaud et al., 2018). Therefore, the possibility that PHGDH inhibition could sensitize osteosarcoma cell lines to cell death by an mTORC1 inhibitor was explored. Treatment with rapamycin caused, as previously demonstrated, a decrease in proliferation in osteosarcoma (Figure 5A) (Zhao et al., 2015). However, combining increasing concentrations of rapamycin and NCT-503 caused no significant cell death (Figures 5B and S5A).

Previously, etomoxir and perhexiline, two inhibitors of CPT1/2 and beta-oxidation, were tested to demonstrate their effect on fatty acid oxidation and cell viability in osteosarcoma cell lines. Neither CPT1 inhibitor caused a change in oxygen consumption rate, even when cells were exposed to up to eight times the working concentration of etomoxir or four times the working concentration of perhexiline (Figures S5B and S5C). Furthermore, etomoxir alone or in combination with NCT-503 had no cytostatic or cytotoxic effects (Figure S5D). Based on the specificity of etomoxir as a CPT1/2 inhibitor and the known non-rapalog mTORC1 inhibitory capacity of perhexiline at lower doses (Balgi et al., 2009), lower concentrations of perhexiline were also tested as mTOR inhibitors.

Levels of total RSP6, a downstream target of mTOR, were significantly decreased by perhexiline, as well as rapamycin (Figure 5C). Additionally, total mTOR expression was found to decrease with perhexiline treatment (Figures S5E and S5F). Indeed, perhexiline treatment at relatively low concentrations (10 μM) did inhibit cellular proliferation (Figure 5D) and cause cell death (Figure 5E). At lower concentrations (5 μM), perhexiline as a single agent did not inhibit proliferation (Figure 5D), but in combination with NCT-503 was found to cause significant cell death in NOS1 (Figure 5E), as well as Saos2 and U2OS (Figures S5G and S5H). This phenotype was also shown with PKUMDL-WQ-2101 combined with perhexiline, showing that this effect was on target with PHGDH inhibition (Figure 5F). CalcuSyn analysis showed that the effects of perhexiline in combination with NCT-503 were synergistic (Figure 5G). Furthermore, the cytotoxic combination was active in other PHGDH-high (Figure 5H), but not PHGDH-low (Figure 5I) or PHGDH knockdown (Figure S5I) cell lines.

Finally, to test the efficacy of this dual inhibition *in vivo*, U2OS cells were xenografted onto the right flank of athymic nude mice, and mice were treated daily with vehicle, NCT-503, perhexiline, or a combination of NCT-503 and perhexiline. Single-agent NCT-503 was not able to slow tumor growth when compared with the vehicle. Single-agent perhexiline had a moderate effect on tumor growth rate. The combination of NCT-503 and perhexiline had a significant and marked effect on U2OS xenografts *in vivo*, resulting in sustained inhibition of tumor growth over 30 days (Figures 5J and S5J).

### Accumulation of SAM and methionine contribute to activation of the GATOR pathway, suggesting an mTORC1 inhibitory mechanism of perhexiline

**Figure 6.**
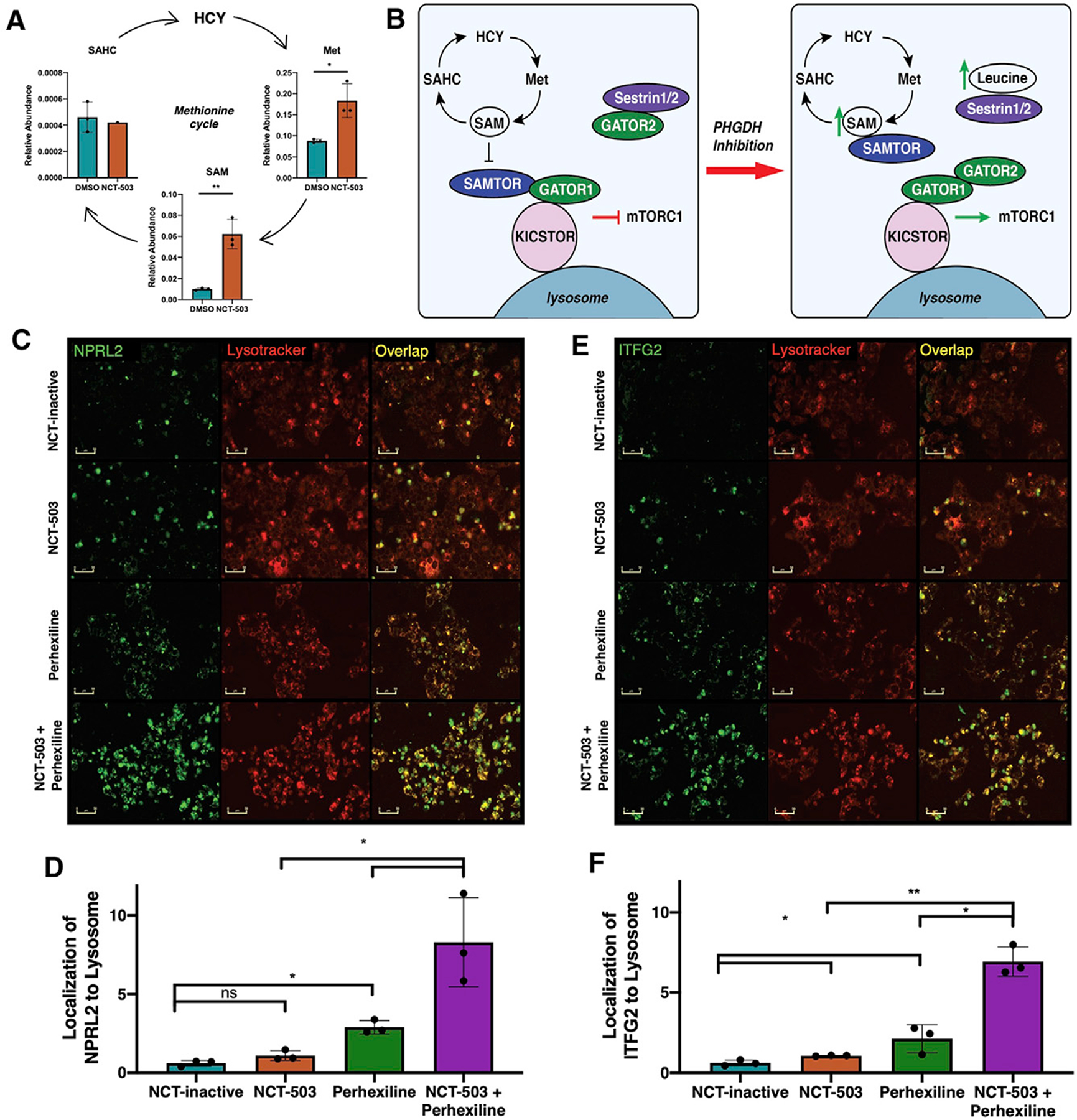
Accumulation of SAM and methionine contribute to activation of GATOR pathway, suggesting mTORC1 inhibitory mechanism of perhexiline (A) Levels of methionine cycle metabolites methionine (Met), *S*-adenosyl methionine (SAM), and *S*-adenosyl homocysteine (SAHC) in NOS1 cells treated with DMSO (vehicle control) or NCT-503 for 48 h. (B) Schematic of effect of PHGDH inhibition on SAM and leucine metabolite levels, downstream mTORC1 pathway activation. (C) Images of NOS1 cells labeled with fluorescent antibody against NPRL2 (GATOR1) (green), fluorescent Lysotracker dye (red), and overlay of green and red showing NPRL2 localization at lysosome (yellow) in various conditions: NCT-inactive, NCT-503, perhexiline, and NCT-503 combined with perhexiline for 48 h. Scale bars represent 50 μm. (D) Quantification of overlap of NPRL2 antibody with Lysotracker, normalized to cell count. (E) Images of NOS1 cells labeled with fluorescent antibody against ITFG2 (KICSTOR) (green), fluorescent Lysotracker dye (red), and overlay of green and red showing ITFG2 localization at lysosome (yellow) in various conditions: NCT-inactive, NCT-503, perhexiline, and NCT-503 combined with perhexiline for 48 h. Scale bars represent 50 μm. (F) Quantification of overlap of ITFG2 antibody with Lysotracker, normalized to cell count. Bars represent means of values; error bars represent SEM. All assays were conducted with n = 3 replicates. *p < 0.05, **p < 0.01, ***p < 0.005, ****p < 0.001.

Given the differential mTORC1 inhibitory activity of perhexiline and rapamycin and the limited literature regarding perhexiline as an mTORC1 inhibitor, the mechanism by which perhexiline inhibits the mTORC1 signaling pathway was explored. Metabolomic analyses of pathways involved in mTORC1 regulation showed that PHGDH inhibition caused an accumulation of intermediates of the methionine cycle, including methionine (Met) and *S*-adenosylmethionine (SAM) (Figure 6A). As methionine synthesis requires a one-carbon unit from the folate cycle, a lack of flux through the folate cycle would also inhibit flux through the methionine cycle, as shown by the lack of change in *S*-adenosyl homocysteine (SAHC) (Figure 6A).

SAM accumulation has been implicated in the activation of mTOR through the SAMTOR-GATOR1 interaction (Gu et al., 2017). The GATOR1 complex is an mTORC1 inhibitory complex when bound to the lysosome through recruitment by the KICSTOR protein complex (Wolfson et al., 2017). When bound to SAM, the SAMTOR protein dissociates from the KICSTOR-GATOR1 complex, inhibiting GATOR1 and allowing for mTORC1 activation (Figure 6B). Furthermore, elevated leucine can bind to Sestrin, releasing the GATOR2 complex that further inhibits GATOR1 (Kim et al., 2015). Given the combined metabolite data implicating the activation of this pathway with PHGDH inhibition, the effects of perhexiline on the KICSTOR-GATOR pathway were explored.

Real-time fluorescent antibody labeling of NPRL2 (lysosome-binding component of GATOR1) and lysosomes (Figure 6C) by IncuCyte S3 live-cell analysis demonstrated that there was significantly increased NPRL2 localization at the lysosome with perhexiline treatment in NOS1, which was further enhanced with the combination treatment of NCT-503 and perhexiline (Figures 6D). Similarly, labeling of ITFG2 (the lysosome-binding component of KICSTOR) and the lysosome (Figure 6E) showed that this localization was also significantly increased with perhexiline treatment in NOS1, and further enhanced with the combination (Figure 6F). Importantly, this localization was found to hold in the PHGDH-low breast cancer MDA-MB-231 as well (Figure S5), demonstrating that the mechanism of increased localization of KICSTOR-GATOR1 to the lysosome by perhexiline is not PHGDH-dependent. Taken together, these findings suggest a possible mechanism of mTORC1 inhibition by perhexiline beyond decreased mTORC1 protein expression, in which perhexiline treatment increases localization of mTORC1-inhibiting sensor complexes to the lysosome.

## DISCUSSION

High-dose methotrexate has been part of the standard-of-care in osteosarcoma for more than 40 years. Due to toxicity, identification of therapies that could more-specifically target the cellular phenotype of osteosarcoma is of high clinical importance. As high doses of methotrexate are required to see anti-proliferative effects *in vitro*, we predicted that osteosarcoma likely depended on a metabolic pathway related to, but not directly involved with, the folate cycle.

PHGDH has been demonstrated to be highly expressed in a wide variety of cancers. Preferential use of the serine biosynthetic pathway offers an increased capacity for cancer cells to generate biomass, facilitating rapid growth and cell division. The increased levels of PHGDH in cancerous tissue offer a metabolic target for treatments with a high therapeutic index. Our finding that high levels of PHGDH correlate with poorer overall survival is of interest as a prognostic marker and potential predictive biomarker and corroborates other studies that have shown increased PHGDH to be characteristic of more-aggressive biology (Jia et al., 2016; Ma et al., 2019; Mullarky et al., 2011; Xian et al., 2016). Furthermore, PHGDH has been shown to be negatively regulated by wild-type p53 in melanoma (Ou et al., 2015). A study from St. Jude and MD Anderson found that more than 90% of surveyed patients with osteosarcoma had some alteration in p53, suggesting a possible mechanism for PHGDH overexpression in p53 mutant cancers (Chen et al., 2014). PHGDH could, therefore, be explored as a biomarker in other tumor types, as high expression may predict for treatment response.

PHGDH inhibition only causes a decrease in cellular proliferation, unless paired with serine- and glycine-deprived conditions or very high concentrations of inhibitor, which are likely not feasible for treating patients (Chen et al., 2013; Mullarky et al., 2016). In osteosarcoma, depriving cells of serine and glycine did not change the efficacy of PHGDH inhibition (Figures 2E and 2F), demonstrating that osteosarcoma may be solely reliant on *de novo* serine biosynthesis for cell survival and suggesting that extracellular serine- and glycine-indifference may be unique to bone tumors. RNA-seq data from the Cancer Cell Line Encyclopedia (CCLE; Broad Institute) indicated relatively low-baseline mRNA expression of the serine transporter ASCT2/SLC1A5, which could explain why PHGDH inhibition cannot be compensated for with extracellular serine and glycine in osteosarcoma cells (Barretina et al., 2012). This could also be due to the biology of the bone microenvironment, which has been shown to benefit from cells that have endogenous serine production (Pollari et al., 2011). Delving deeper, the metabolomic implications of PHGDH inhibition show a complex regulatory network that integrates not only serine and glycine but also other amino acids, nucleotides, mitochondrial respiration, and molecular signaling pathways that promote cellular survival. Importantly, because PHGDH inhibition does result in decreased cell proliferation, *de novo* serine biosynthesis does provide some of the nutrients required for biomass production and proliferation but does not encompass the entire survival capacity of osteosarcoma.

The entry of a one-carbon unit from serine into the folate cycle links serine biosynthesis to one-carbon metabolism, which incorporates the folate cycle and methionine cycle. These metabolic pathways regulate amino acid synthesis, purine and pyrimidine metabolism, SAM levels, and methylation capacity, as well as the maintenance of redox homeostasis through glutathione (GSH), ATP, and NADH generation (Shuvalov et al., 2017), making upregulation of this system critical for the generation of biomass in cancer cells.

The independent metabolomics and [U-^13^C] glucose tracings conducted in osteosarcoma cell lines demonstrated that PHGDH inhibition consistently modified these pathways. Both metabolomics analyses identified increased glucose flux through glycolysis and decreased TCA cycle activity with NCT-503 treatment (Figures 2 and S2). Critical to the effect of PHGDH inhibition on the TCA cycle is the contribution of *de novo* serine biosynthesis to total alpha-ketoglutarate (αKG). The serine biosynthetic enzyme PSAT1 converts 3PP into 3PSer. The same enzymatic reaction converts glutamate to αKG (Fan et al., 2015). Glutaminolysis and isocitrate dehydrogenation provide additional sources of αKG to the cell, and the unlabeled αKG present with PHGDH inhibition validates the contribution of other cellular pathways to the αKG pool in the absence of *de novo* serine biosynthesis (Maus and Peters, 2017).

The accumulation of fatty acids because of PHGDH inhibition was another metabolic phenotype of interest. Fatty acids were found to likely come from decreased mitochondrial activity, rather than from fatty acid synthesis by SREBP1 or extracellular uptake (Figures S3F and S3G). Given the high levels of *de novo* serine biosynthesis in PHGDH-high osteosarcoma, the basal flux of glucose would likely be through the serine synthetic pathway, necessitating alternate mitochondrial fuel sources, such as fatty acids. Glutamine metabolism likely did not contribute to mitochondrial oxidative phosphorylation (Figures S3A–S3C), and the role of baseline and adaptive glutamine metabolism are the subjects of further research. However, [U-^13^C] palmitate tracing did demonstrate incorporation of fatty acids to downstream metabolites of acetyl-coA with NCT-503 treatment, suggesting a basal capacity to use fatty acids (Figures S3D and S3E). With net TCA cycle inhibition by NCT-503, however, the fatty acid fuel source accumulated and could contribute to mTORC1 activation via lipid sensing pathways (Menon et al., 2017).

Similarly, PHGDH inhibition also resulted in accumulation of BCAAs (Figures 4I–4K), which further contribute to activation of the mTORC1 pathway (Wolfson et al., 2016). PHGDH inhibition drove increased gene expression of *SLC3A2* and *SLC7A5* (Figure S4). These two amino acid transporters drive the transport of leucine into the lysosome, resulting in mTORC1 activation (Milkereit et al., 2015). It is important to note that the inhibition of *de novo* serine biosynthesis by NCT-503 naturally results in decreased incorporation of imported glucose into intracellular serine and glycine (Figures 2J and 2K). This decrease in amino acid levels could theoretically inhibit mTORC1 activity, triggering nutrient sensors that would activate autophagy to increase serine and glycine levels. However, the accumulation of BCAAs in response to PHGDH inhibition seems to override serine and glycine signaling to mTOR, enhancing mTORC1 activation. The genetic response to small-molecule inhibition also showed increased expression of serine biosynthetic enzymes *PHGDH* and *PSAT1*, as well as *ATF4* (Figure 4), which has been associated with transcriptional serine biosynthetic pathway regulation (Zhao et al., 2016). Importantly, mTORC1 has been shown to activate *ATF4* in response to cellular requirements for purine synthesis (Ben-Sahra et al., 2016), a metabolic consequence of PHGDH inhibition.

The activation of mTORC1 has been demonstrated to increase oxidative phosphorylation in cancer cells, primarily through the regulation of metabolic genes such as SREBP-1 and ATP synthase components (de la Cruz López et al., 2019). Although the metabolomics analyses conducted did not demonstrate induction of oxidative phosphorylation by mTORC1, it is likely that the inhibition of oxygen consumption by NCT-503 treatment is the dominant pathway in this system (Manifava et al., 2016). Similarly, BCAA catabolism has been demonstrated to supplement the TCA cycle; however, BCAA catabolism requires αKG to produce branched-chain keto acids, which then enter the TCA cycle (Holeček, 2018). Because αKG is depleted in cells treated with NCT-503, BCAA catabolism is likely not a dominant cell-survival pathway in response to PHGDH inhibition.

Prior work from our laboratory has shown that single-agent metabolic therapies are rarely effective in a clinical setting (Kremer et al., 2017; Prudner et al., 2019; Robinson et al., 2019). Given that PHGDH inhibition results in metabolite and lipid accumulation that activated mTORC1 from multiple pathways, we hypothesized that NCT-503 could sensitize to mTORC1 inhibition. Previous studies have observed that PHGDH inhibition induced autophagy in carcinoma cells but have attributed that effect to an mTOR-independent mechanism because of the lack of response to rapamycin treatment (Mita and Tolcher, 2007; Sharif et al., 2018). The lack of rapamycin activity in this system correlates with literature demonstrating that mTOR inhibitors combined with other agents have had limited clinical efficacy in osteosarcoma (Fleuren et al., 2014; Martin-Broto et al., 2017).

The implications of NCT-503 sensitization to non-rapalog mTORC1 inhibition offer a solution to previous negative clinical trials using mTOR inhibitors in osteosarcoma. High PHGDH expression has been identified as a critical driver of sorafenib resistance, suggesting that PHGDH biology can drive efficacy of mTOR inhibition (Wei et al., 2019). The significant effects of NCT-503 combined with perhexiline *in vivo*, with minimal toxicity and dramatic tumor control, show that PHGDH inhibition combined with mTORC1-signaling inhibition are a promising dual therapy in PHGDH-high cancers. Additionally, because perhexiline is already used in the clinic, this drug has the clinical potential to be re-purposed as an mTORC1-inhibiting cancer therapeutic (Beadle et al., 2015).

Although the primary use of perhexiline is as a CPT1/2 inhibitor, the half-maximal inhibitory concentration IC_50_ for these effects ranges from 70 to 150 μM (Kennedy et al., 2000). At lower concentrations (10 μM), perhexiline has been shown to have anti-mTORC1 activity (Balgi et al., 2009). Identifying the mechanism of perhexiline as a non-rapalog mTORC1-signaling inhibitor classifies perhexiline as a metabolic signaling inhibitor with potential in cancer therapy. As the combination with NCT-503 was significantly more cytotoxic with perhexiline, rather than rapamycin, this mechanism is likely related to NCT-503-driven metabolite-accumulation changes. The accumulation of BCAAs, SAM, and Met implicates GATOR biology. The accumulation of SAM and Met was expected with PHGDH inhibition because NCT-503 treatment would result in decreased folate-cycle activity. The enzyme MTHFR converts 5,10-methylenetetrahydrofolate into 5-methyltetrahydrofolate, which then enters the methionine cycle under normal conditions. When the methionine cycle is blocked as a downstream result of PHGDH inhibition, SAM and methionine accumulate.

The protein complex GATOR1, consisting of NPRL2, NPRL3, and DEPDC5, has been shown to be an inhibitor of mTORC1 when recruited to the lysosome by KICSTOR (Wolfson et al., 2017). The GATOR2 protein complex also regulates mTORC1 by inhibiting GATOR1 (Kim et al., 2015). Upon PHGDH inhibition, the accumulation of SAM supports the SAMTOR-mediated inhibition of GATOR1, whereas the accumulation of BCAAs, and specifically leucine, support Sestrin-1/2-mediated GATOR2 activation. The effects of perhexiline treatment on GATOR1 and KICSTOR localization to the lysosome offer a potential mechanism for mTORC1 inhibition. The increased localization of KICSTOR and GATOR1 to the lysosome drives the inhibition of mTOR and provides a mechanism of inhibition that is unique from rapamycin. A more complete understanding of perhexiline as an mTORC1 pathway inhibitor is, therefore, needed.

Given the significantly shorter overall survival in patients with high PHGDH expression, there is likely a large subset of patients with osteosarcoma that do not benefit from exposure to high-dose methotrexate. PHGDH should therefore be explored as a prognostic and predictive biomarker of an ineffective and potentially toxic treatment. A subset of patients could be considered for alternative approaches that target PHGDH expression via a combination therapy that could replace toxic high-dose methotrexate. Our data showing the significant tumor-control potential of the combination of NCT-503 and perhexiline *in vivo* emphasize the importance of repurposing perhexiline for oncology purposes. This work, therefore, provides the preclinical rationale for testing a metabolic combination therapy targeting PHGDH and mTORC1 biology as a replacement for high-dose methotrexate in osteosarcoma.

## STAR★METHODS

### RESOURCE AVAILABILITY

#### Lead contact

Further information and requests for resources and reagents should be directed to and will be fulfilled by the Lead Contact, Brian Van Tine (bvantine@wustl.edu)

#### Materials availability

This study did not generate new unique reagents.

#### Data and code availability

This study did not generate or analyze unique code. Original data for metabolomics and lipidomics are available as Tables S2 and S3.

### EXPERIMENTAL MODEL AND SUBJECT DETAILS

#### Animal studies

Mouse xenograft protocols were approved by Washington University in St. Louis Institutional Animal Care and Use Committee (IACUC). Mice were maintained under IACUC guidelines. Mice were randomly assigned to treatment groups as xenograft-derived tumors reached an average volume of 200 mm^3^. Investigators were not blind to treatment groups. 2 × 10^6^ U2OS cells were collected in 75 μL of DMEM and combined with 75 μL of Matrigel (Cat # 354234, Corning, Corning, NY), and grafted by subcutaneous injection into the right flank of athymic nude mice (female, 4–6 weeks old, homozygous for Foxn1^nu^, Cat # 002019, Jackson Laboratories, Bar Harbor, ME). Length and width of resulting tumors from xenograft were measured daily, and tumor volumes were calculated using the formula (length × width^2^)/2. Cell line-derived xenografts were allowed to grow to an average volume of 200 mm^3^, at which point mice were randomly assigned to treatment groups of either vehicle (5% ethanol, 35% polyethylene glycol 300 (Cat # 8.17002, Sigma Aldrich, St. Louis, MO), 60% aqueous 30% hydroxyropyl-beta-cyclodextrin (Cat # H107, Sigma Aldrich, St. Louis, MO)) daily, 40 mg/kg NCT-503 (in vehicle) intraperitoneally (IP) daily, 8 mg/kg perhexiline (in 1.8% w/v hydroxypropyl-beta-cyclodextrin) by oral gavage daily, or 40 mg/kg NCT-503 IP and 8 mg/kg perhexiline by oral gavage daily. Tumors were allowed to grow to volumes of 2000 mm^3^ or until 30 days had passed. Ten mice per treatment group were utilized for all studies. Tumor growth curves are shown as mean ± SEM, with variance across groups compared for statistical significance by two-way ANOVA. Asterisks represent p values: * < 0.05, ** < 0.01, *** < 0.005, **** < 0.001.

#### Cell lines

NOS1, Saos2, U2OS, and MNNG were cultured in MEM (Cat # 11095072, GIBCO, Thermo Fisher, Waltham, MA) supplemented with 10% FBS (Cat # S11150, R&D Systems, Bio-Techne, Minneapolis, MN), 1.3% penicillin/streptomycin (Cat # 15140122, GIBCO, Thermo Fisher, Waltham, MA), 286 μM serine (Cat # AAJ6218709, Fisher Scientific, Hampton, NH) and 286 μM glycine (Cat # 67419, Sigma-Aldrich, St. Louis, MO). MG63, MDA-MB-231, and MDA-MB-468 were cultured in DMEM (Cat # 11965084, GIBCO, Thermo Fisher, Waltham, MA) supplemented with 10% FBS and 1.3% penicillin/streptomycin. All cells were maintained at 37°C and 5% CO_2_ in a humidified atmosphere. All cells were cultured in prophylactic plasmocin (Cat # ant-mpp, InvivoGen, San Diego, CA) and checked regularly for mycoplasma contamination.

### METHOD DETAILS

#### NucRed and shPHGDH cell line generation

All cell lines were transduced with IncuCyte NucLight Red lentivirus reagent (EF-1 Alpha promoter, Puromycin selection) (Cat # 4476, Sartorius, Ann Arbor, MI, USA). Briefly, cells were seeded and allowed to adhere for 24 h to reach approximately 20%–40% confluence. NucLight reagent was diluted in DMEM containing 8 μg/mL polybrene (Cat # sc-134220, Santa Cruz Biotechnology, Dallas, TX) and added to cells for 48 h. Media was then replaced with fresh DMEM containing 3 μg/mL puromycin (Cat # MIR 5940, Mirus Bio, Madison, WI). Red fluorescence was monitored on the IncuCyte ZOOM system (Sartorius, Ann Arbor, MI), and analyzed using the IncuCyte image analysis software (Sartorius, Ann Arbor, MI). Similarly, shPHGDH cells were generated using lentiviral transduction particles (shPHGDH_1, TRCN0000028520, Sigma Aldrich, St. Louis, MO, USA; shPHGDH_2, TRCN0000233033, Sigma Aldrich, St. Louis, MO, USA). shGFP vectors were utilized as transduction controls. Knockdown of PHGDH was validated using the Wes Automated Western Blotting System (ProteinSimple, Bio-Techne, Minneapolis, MN), which uses automated capillary technology to measure protein expression and total protein concentration, per manufacturer’s protocol.

#### Proliferation and cell death assays

NucRed cells were seeded in 96 well tissue-culture plates (Cat # TP92096, Midwest Scientific, Valley Park, MO). At time of treatment, culture media was removed from wells, and was replaced with treatment condition of interest, diluted in phenol red-free MEM (Cat # 51200038, GIBCO, Thermo Fisher, Waltham, MA) supplemented with 10% FBS or 10% dialyzed FBS (Cat # A3382001, GIBCO, Thermo Fisher, Waltham, MA) or 10% lipoprotein-depleted FBS (Cat # 880100–5, Kalen Biomedical, Germantown, MD), 2 mM L-glutamine (Cat # 25030081, GIBCO, Thermo Fisher, Waltham, MA), 286 μM serine, and 286 μM glycine. Red nuclear fluorescence was monitored on the IncuCyte ZOOM system and analyzed using the IncuCyte image analysis software. Proliferation was normalized to red count/mm^2^ at starting time point. Cell death assays were conducted in a similar way, with treatment media including 50 nM YOYO-1 Iodide (Cat # Y3601, Invitrogen, Carlsbad, CA). Green YOYO-1 iodide fluorescence and red nuclear fluorescence were monitored on the IncuCyte ZOOM system and analyzed using the IncuCyte image analysis software. Cell death was quantified by normalizing green count/mm^2^ to red count/mm^2^ to account for differences in cell density, and then normalized to starting time point. Percent cell death was calculated using a 100% death endpoint measurement. CalcuSyn software was utilized to measure combination index for synergy assays. Quantification of analyses are summarized as mean ± SEM, with variance across groups compared for statistical significance by Student’s t test on Prism 8 software (GraphPad Software, San Diego, CA). Asterisks represent p values: * < 0.05, ** < 0.01, *** < 0.005, **** < 0.001.

#### Immunohistochemistry

Tumor microarray of osteosarcoma patient samples was obtained from the University of Texas MD Anderson Cancer Center. Immunohistochemistry was conducted using anti-PHGDH antibody. Briefly, slides were baked at 50°C for 2 h. Slides were then deparaffinized in xylene (Cat # 247642, Sigma-Aldrich, St. Louis, MO) (2 × 5 min) and rehydrated with 100% ethanol (EtOH) (Cat # A4094, Fisher Scientific, Hampton, NH) (2 × 2.5 min), 95% EtOH (2 × 2 min), and 75% EtOH (1 min). Endogenous peroxidase was blocked using 3% hydrogen peroxide, made from 30% hydrogen peroxide (Cat # 216763, Sigma-Aldrich, St. Louis, MO) diluted in 1X PBS (Cat # 14190136, GIBCO, Thermo Fisher, Waltham, MA). Antigen unmasking was conducted using citrate-based antigen unmasking solution (Cat # H-3300–250, Vector Laboratories, Burlingame, CA). Blocking was conducted using blocking buffer (3% w/v BSA (Cat # A9418, Sigma-Aldrich, St. Louis) in 1X PBS). Primary antibody for PHGDH (anti-rabbit, Cat # HPA021241, Sigma-Aldrich, St. Louis, MO) was diluted 1:1000 in blocking buffer. Slides were then washed 3 × 5 min with 1X Tris-Buffered Saline (Cat # T5912, Sigma-Aldrich, St. Louis, MO) with 0.1% Tween 20 Detergent (Cat # P9416, Sigma-Aldrich, St. Louis, MO) (TBST). Secondary anti-rabbit antibody (Cat # 211-032-171, Jackson ImmunoResearch, West Grove, PA) was diluted 1:1000 in blocking buffer. Hematoxylin/eosin were used to counterstain. Slides were mounted using anti-fade mounting media (Cat # P36934, Invitrogen, Carlsbad, CA) and visualized using an Olympus BX51 fluorescence microscope (Olympus Life Science, Waltham, MA) by one party. A second party blind scored the slides. A third party correlated the blind scoring to patient data. RNA sequencing data with available outcomes data for the second TMA was obtained from the University of Texas MD Anderson Cancer Center (Wu et al., 2020).

#### Immuno assays

Lysates were obtained for all cell lines by collecting 2 × 10^6^ cells and washing three times with 1X PBS. Cells were then pelleted and lysed per reagent protocol for cell lysis buffer (Cat # 9803, Cell Signaling Technology, Danvers, MA) with supplemented protease and phosphatase inhibitors (Cat # 78440, Thermo Scientific, Waltham, MA). Protein concentration within cell lysates were determined using Bradford Assay (Cat # 5000202, Bio-Rad, Hercules, CA). Protein expression for PHGDH was analyzed using the Wes Automated Western Blotting System (ProteinSimple, Bio-Techne, Minneapolis, MN), which uses automated capillary technology to measure protein expression and total protein concentration, per manufacturer’s protocol. PHGDH protein expression was detected using 12–230 kDa Separation Modules. Anti-PHGDH antibody (anti-rabbit, Cat # HPA021241, Sigma-Aldrich, St. Louis, MO) was diluted 1:100 for signal detection. Anti-SREBP1 antibody (anti-mouse, Cat # sc-13551, Santa Cruz Biotechnology, Dallas, TX) was diluted 1:50 for signal detection. Anti-mTOR antibody (anti-rabbit, Cat # 2972, Cell Signaling Technology, Danvers, MA) was diluted 1:50 for signal detection. Anti-phospho p70 S6 Kinase (Thr389) antibody (anti-rabbit, Cat # 9205, Cell Signaling Technology, Danvers, MA) was diluted 1:50 for signal detection. Anti-p70 S6 Kinase antibody (anti-rabbit, Cat # 9202, Cell Signaling Technology, Danvers, MA) was diluted 1:50 for signal detection. Protein levels were visualized and quantified using Compass for SimpleWestern software (ProteinSimple, Bio-Techne, Minneapolis, MN). Quantification of analyses are summarized as mean ± SEM, with variance across groups compared for statistical significance by Student’s t test. Asterisks represent p values: * < 0.05, ** < 0.01, *** < 0.005, **** < 0.001.

#### Seahorse metabolomics analysis

Cells were plated in Seahorse XF96 Cell Culture Microplates (Cat # 101085–004, Agilent Technologies, Santa Clara, CA). Seeding density was optimized for Seahorse based on recommended ranges of oxygen consumption rate (OCR) and extracellular acidification rate (ECAR) for baseline data (Seahorse XF Assay Basic Procedures). Basal mitochondrial respiration was measured using the Seahorse XF Cell Mito Stress Test Kit (Cat # 103015–100, Agilent Technologies, Santa Clara, CA) per kit protocol. Basal glycolytic rate and glycolytic proton efflux rate were measured using the Seahorse XF Glycolytic Rate Test Kit (Cat # 103710–100, Agilent Technologies, Santa Clara, CA) per kit protocol. Mitochondrial fuel source flexibility was measured using the Seahorse XF Mito Fuel Flex Test Kit (Cat # 103260–100, Agilent Technologies, Santa Clara, CA) per kit protocol. All assays were run on a Seahorse XFe96 Analyzer (Agilent Technologies, Santa Clara, CA), and data were quantified and reported using Seahorse Wave Desktop Software (Agilent Technologies, Santa Clara, CA) automated reports. Quantification of analyses are summarized as mean ± SEM, with variance across groups compared for statistical significance by Student’s t test. Asterisks represent p values: * < 0.05, ** < 0.01, *** < 0.005, **** < 0.001.

#### Intracellular metabolomics analysis

NOS1 cells were plated at 8 × 10^5^ cells per dish in 10 cm dishes and treated with either DMSO or 15 μM NCT-503, diluted in MEM containing 10% FBS, 286 μM serine, and 286 μM glycine for 48 h. Methanol metabolite extraction was performed according to the Human Metabolome Technologies (HMT) Metabolomics Analysis Sample Preparation Protocol method for adherent cells (Human Metabolome Technologies, Boston, MA). Metabolite concentrations were normalized to cell count. Samples were submitted in triplicate. Quantification of analyses are summarized as mean ± SEM, with variance across groups compared for statistical significance by Student’s t test. Pathway analysis of metabolites was conducted using MetaboAnalyst 4.0 (Montreal, Quebec, Canada). Data were glog transformed and Pareto scaled, and p values were adjusted (Chong et al., 2019). Asterisks represent p values: * < 0.05, ** < 0.01, *** < 0.005, **** < 0.001.

#### Intracellular [U-^13^C] tracing analysis

Saos2 cells were plated at 2.8 × 10^6^ cells per dish in 10 cm dishes and allowed to adhere for 24 h. Prior to experiment, dialyzed FBS was complexed with palmitate (Cat # P0500–25G, Sigma Aldrich, St. Louis, MO) or [U-^13^C_16_] palmitate (Cat # CLM-409-PK, Cambridge Isotope Laboratories, Tewksbury, MA) by rocking at 37°C overnight (Kamphorst et al., 2014). Cells were treated with either 10 μM NCT-inactive or 15 μM NCT-503, diluted in DMEM containing 5.5 mM glucose, 2 mM glutamine, and 10% FBS for 21 h to reach steady state. Media was then replaced with fresh media containing drug for 3 h. Media was then replaced with fresh media containing dialyzed FBS supplemented with palmitate and [U-^13^C] glucose (Cat # CLM-1396–0.5, Cambridge Isotope Laboratories, Tewksbury, MA) for glucose tracing, or with [U-^13^C] palmitate and unlabeled glucose for palmitate tracing. Cells were maintained in labeled medium for 24 h (Locasale et al., 2011; Reid et al., 2018). Methanol metabolite extraction was performed according to the Human Metabolome Technologies (HMT) Metabolomics Analysis Sample Preparation Protocol method for adherent cells (Human Metabolome Technologies, Boston, MA). Metabolite concentrations were normalized to cell count and [U-^13^C] concentrations were corrected for natural abundance. Samples were submitted in triplicate. Quantification of analyses are summarized as mean ± SEM, with variance across groups compared for statistical significance by Student’s t test. Asterisks represent p values: * < 0.05, ** < 0.01, *** < 0.005, **** < 0.001.

#### BODIPY kinetic assays

For BODIPY fluorescence assays, NucRed cells were seeded in 96 well tissue-culture plates. When at optimal confluence, culture media was removed from wells, and was replaced with media containing BODIPY 493/503 (Cat # D3922, Invitrogen, Carlsbad, CA) diluted 1:1000 in phenol red-free media. Green fluorescence and red nuclear fluorescence were monitored on the IncuCyte ZOOM system and analyzed using the IncuCyte image analysis software. Neutral fatty acid levels were quantified by normalizing green count/well to red count/well to account for differences in cell density, and then normalized to starting time point. Quantification of analyses are summarized as mean ± SEM, with variance across groups compared for statistical significance by two-way ANOVA. Asterisks represent p values: * < 0.05, ** < 0.01, *** < 0.005, **** < 0.001.

#### Lipidomics analysis

Cells were plated at 1 × 10^6^ cells per dish in 10 cm dishes and treated with either 10 μM NCT-inactive or 10 μM NCT-inactive with 15 μM NCT-503 for 48 h. Cells were collected by cell scraping, washed with 1X PBS, and were counted. A total of 2 × 10^6^ cells were submitted to Washington University in St. Louis Diabetic Cardiovascular Disease Center for lipidomics analysis, where analytes were measured by liquid chromatography/mass spectrometry (LC-MS). All data were reported as peak area ratio of analyte relative to internal standard. Samples were submitted in triplicate. Quantification of analyses are summarized as mean ± SEM, with variance across groups compared for statistical significance by Student’s t test. Asterisks represent p values: * < 0.05, ** < 0.01, *** < 0.005, **** < 0.001.

#### NanoString metabolism gene panel

Cells were plated at 1 × 10^6^ cells per dish in 10 cm dishes and treated with either 10 μM NCT-inactive or 10 μM NCT-inactive with 15 μM NCT-503 for 24 h. Cells were then lysed and RNA was collected using the Direct-zol RNA Miniprep Plus Kit (Cat # R2070, Zymo Research, Irvine, CA), and 250 ng of RNA were sent to NanoString; samples were submitted in triplicate. Quantification of analyses are summarized as mean ± SEM, with variance across groups compared for statistical significance by Student’s t test. Asterisks represent p values: * < 0.05, ** < 0.01, *** < 0.005, **** < 0.001.

#### MilliPlex MAP magnetic bead-based assay

Cells were plated in 6-well plates. When confluent, cells were serum-starved for 4 h. After 4 h, media was replaced with DMEM containing serum and relevant treatment conditions: serum alone (NT), 10 μM NCT-inactive, 5 μM perhexiline, or 10 μM rapamycin. Cells were incubated at 37°C for 24 h. Samples were collected according to manufacturer’s instructions (Cat # 48–612MAG, Millipore, Burlington, MA) and 16 μg of sample were submitted for analysis. Measurements were taken on a Luminex FlexMap 3D system. Quantification of analyses are summarized as mean ± SEM, with variance across groups compared for statistical significance by Student’s t test. Asterisks represent p values: * < 0.05, ** < 0.01, *** < 0.005, **** < 0.001.

#### Activity and metabolite assays

Cells were plated at 3 × 10^6^ cells per dish in 10 cm dishes and treated with either 10 μM NCT-inactive or 10 μM NCT-inactive with 15 μM NCT-503 for 48 h. PHGDH activity was assessed using cell lysates in the PHGDH Activity Assay Kit (Cat # PK-CA577-K569, PromoCell, Heidelberg, Germany) per manufacturer’s protocol. Extracellular lactate levels were assessed using extracellular medium in the PicoProbe Lactate Fluorometric Assay Kit (Cat # K638, Biovision, Milpitas, CA) per manufacturer’s protocol. Total and serine-derived αKG levels were assessed using cell lysates in the Alpha Ketoglutarate (alpha KG) Assay Kit (Cat # ab83431, Abcam, Cambridge, United Kingdom) per manufacturer’s protocol.

#### Lysosome localization assays

For antibody-based fluorescence assays, wild-type cells were seeded in 96 well tissue-culture plates. When at optimal confluence, culture media was removed from wells, and was replaced with phenol red-free media containing relevant treatment conditions, antibody diluted 1:1500, and LysoTracker Deep Red (Cat # L12492, Invitrogen, Thermo Fisher, Waltham, MA) diluted to 50 nM. Antibodies used were anti-ITFG2 (anti-mouse, Cat # sc-271420, Santa Cruz Biotechnology, Dallas, TX) and anti-NPRL2 (anti-mouse, Cat # sc-376986, Santa Cruz Biotechnology, Dallas, TX). Green fluorescence and red fluorescence were monitored on the IncuCyte ZOOM system at 20X magnification and analyzed using the IncuCyte image analysis software. Protein localization to the lysosome was quantified using IncuCyte image analysis software to quantify overlap of green and red, and normalizing this metric to cell density, which was then normalized to starting time point. Quantification of analyses are summarized as mean ± SEM, with variance across groups compared for statistical significance by Student’s t test. Asterisks represent p values: * < 0.05, ** < 0.01, *** < 0.005, **** < 0.001.

### QUANTIFICATION AND STATISTICAL ANALYSIS

Statistical details of experiments, including sample size, can be found in the figure legends for each experiment and in the method details for each experiment. Briefly, all data are presented as mean ± SEM, with n = 3 unless otherwise stated, and variance across groups compared for statistical significance by Student’s t test or two-way ANOVA. For the pathway analysis of metabolites measured using intracellular metabolomics, data were glog transformed and Pareto scaled, and p values were adjusted. Grubb’s test was utilized to exclude extreme studentized deviate-based outliers. Asterisks represent p values: * < 0.05, ** < 0.01, *** < 0.005, **** < 0.001.

## Supplementary Material

1

2

3

4

5

## Figures and Tables

**Table:** KEY RESOURCES TABLE

REAGENT or RESOURCE	SOURCE	IDENTIFIER
Antibodies		
Anti-PHGDH polyclonal antibody produced in rabbit	Sigma-Aldrich	Cat# HPA021241; RRID: AB_1855299
Anti-SREBP-1 (2A4) monoclonal antibody produced in mouse	Santa Cruz Biotechnology	Cat# sc-13551; RRID: AB_628282
Anti-mTOR antibody produced in rabbit, unconjugated	Cell Signaling Technology	Cat# 2972; RRID: AB_330978
Anti-Phospho-p70 S6 Kinase (Thr389) polyclonal antibody produced in rabbit	Cell Signaling Technology	Cat# 9205; RRID: AB_330944
Anti-p70 S6 Kinase polyclonal antibody produced in rabbit	Cell Signaling Technology	Cat# 9202; RRID: AB_331676
Anti-ITFG2 (F-11) monoclonal antibody produced in mouse, conjugated to AlexaFluor 488	Santa Cruz Biotechnology	Cat# sc-271420; RRID: AB_10611069
Anti-NPRL2 (F-3) monoclonal antibody produced in mouse, conjugated to AlexaFluor 488	Santa Cruz Biotechnology	Cat# sc-376986
Peroxidase IgG Fraction Monoclonal Mouse Anti-Rabbit IgG, light chain specific antibody	Jackson ImmunoResearch Labs	Cat# 211-032-171; RRID: AB_2339149
Bacterial and virus strains		
PHGDH MISSION shRNA Bacterial Glycerol Stock	Sigma-Aldrich	SHCLNG-NM_006623 TRCN0000028520
PHGDH MISSION shRNA Bacterial Glycerol Stock	Sigma-Aldrich	SHCLNG-NM_006623 TRCN0000233033
MISSION® pLKO.1-puro TurboGFP shRNA Control Transduction Particles	Sigma-Aldrich	SHC004V
Incucyte® NucLight Red Lentivirus Reagent (EF-1 Alpha Promoter, Puromycin selection)	Sartorius	Cat# 4476
Biological samples		
Osteosarcoma tumor microarray	University of Texas MD Anderson Cancer Center	University of Texas MD Anderson Cancer Center
Chemicals, peptides, and recombinant proteins		
Alfa Aesar L-Serine, Cell Culture Reagent	Fisher Scientific	Cat# AAJ6218709
Glycine 1 M solution	Sigma-Aldrich	Cat# 67419
Methotrexate	Cayman Chemical	Cat# 13960
NCT-503 Inactive Control	Sigma-Aldrich	Cat# SML1671
NCT-503	Sigma-Aldrich	Cat# SML1659
PKUMDL-WQ-2101	Sigma-Aldrich	Cat# SML1970
BPTES	Sigma-Aldrich	Cat# SML0601
Rapamycin	Sigma-Aldrich	Cat# R8781
Perhexiline maleate salt	Sigma-Aldrich	Cat# SML0120
InSolution Etomoxir - CAS 828934-41-4 - Calbiochem	Sigma-Aldrich	Cat# 5.09455
Fetal Bovine Serum, dialyzed, US origin, One Shot format	GIBCO, Thermo Fisher Scientific	Cat# A3382001
Lipoprotein Depleted Fetal Bovine Serum	Kalen Biomedical	Cat# 880100-5
YOYO-1 Iodide (491/509) −1 mM Solution in DMSO	Invitrogen	Cat# Y3601
Palmitic acid	Sigma-Aldrich	Cat# P0500-25G
D-Glucose (U-13C6, 99%)	Cambridge Isotope Laboratories	Cat# CLM-1396-0.5
Palmitic Acid (U-13C16, 98%)	Cambridge Isotope Laboratories	Cat# CLM-409-PK
BODIPY 493/503 (4,4-Difluoro-1,3,5,7,8-Pentamethyl-4-Bora-3a,4a-Diaza-*s*-Indacene)	Invitrogen	Cat# D3922
Polyethylene glycol 300	Sigma-Aldrich	Cat# 8.17002
(2-Hydroxypropyl)-β-cyclodextrin	Sigma-Aldrich	Cat# H107
LysoTracker Deep Red	Invitrogen	Cat# L12492
Critical commercial assays		
Quick Start Bradford Protein Assay Kit 2	Bio-Rad	Cat# 5000202
Seahorse XF Cell Mito Stress Test Kit	Agilent	Cat# 103015-100
Seahorse XF Glycolytic Rate Test Kit	Agilent	Cat# 103710-100
Seahorse XF Mito Fuel Flex Test Kit	Agilent	Cat# 103260-100
Direct-zol RNA Miniprep Plus Kit	Zymo Research	Cat# R2070
MILLIPLEX MAP Total Akt/mTOR Magnetic Bead 11-Plex Kit - Cell Signaling Multiplex Assay	Millipore	Cat# 48-612MAG
Phosphoglycerate Dehydrogenase (PHGDH) Activity Assay Kit	PromoCell	Cat# PK-CA577-K569
PicoProbe Lactate Fluorometric Assay Kit	BioVision	Cat# K638
Alpha Ketoglutarate (alpha KG) Assay Kit	Abcam	Cat# ab83431
Experimental models: cell lines		
NOS1	RIKEN BioResource Research Center	Cat# RCB1032; RRID: CVCL_1610
Saos2	ATCC	Cat# HTB-85; RRID: CVCL_0548
U2OS	ATCC	Cat# HTB-96; RRID: CVCL_0042
MG63	ATCC	Cat# CRL-1427; RRID: CVCL_0426
MNNG	ATCC	Cat# CRL-1547; RRID: CVCL_0439
MDA-MB-231	ATCC	Cat# CRL-12532; RRID: CVCL_0062
MDA-MB-468	ATCC	Cat# HTB-132; RRID: CVCL_0419
Experimental models: organisms/strains		
Mouse: athymic nude mice (female, 4–6 weeks old, homozygous for Foxn1^nu^)	The Jackson Laboratory	RRID: IMSR_JAX:002019
Software and algorithms		
GraphPad Prism	GraphPad	RRID: SCR_002798
Incucyte® Base Analysis Software	Sartorius	https://www.essenbioscience.com/en/products/software/incucyte-base-software/
IncuCyte® S3 Software	Sartorius	https://www.essenbioscience.com/en/products/software/incucyte-s3-software-v2018b/
CalcuSyn	Biosoft	http://www.biosoft.com/w/calcusyn.htm
Compass for SimpleWestern	proteinsimple	https://www.proteinsimple.com/software_compass_simplewestern.html
Seahorse Wave	Agilent Technologies	RRID: SCR_014526
MetaboAnalyst 4.0 Software	MetaboAnalyst	https://www.metaboanalyst.ca/

## Highlights

- Upregulated PHGDH in osteosarcoma correlates with poor survival
- Inhibiting PHGDH attenuates osteosarcoma proliferation without causing cell death
- TCA cycle blockade and accumulation of metabolites results in mTORC1 activation
- PHGDH inhibition combined with non-rapalog mTORC1 inhibition is synergistic
