# Supplementary material for: Metabolic compensation activates pro-survival mTORC1 signaling upon 3-phosphoglycerate dehydrogenase inhibition in osteosarcoma
Source: Cell Rep. Author manuscript; Available in PMC 2021 Oct 28. (PMC8552368; doi:10.1016/j.celrep.2020.108678)
Supplement: 1 [file NIHMS1748607-supplement-1.pdf]

Cell Reports, Volume *34*

Supplemental Information

Metabolic compensation activates pro-survival mTORC1 signaling upon 3-phosphoglycerate dehydrogenase inhibition in osteosarcoma

Richa Rathore, Katharine E. Caldwell, Charles Schutt, Caitlyn B. Brashears, Bethany C. Prudner, William R. Ehrhardt, Cheuk Hong Leung, Heather Lin, Najat C. Daw, Hannah C. Beird, Abigail Giles, Wei-Lien Wang, Alexander J. Lazar, John S.A. Chrisinger, J. Andrew Livingston, and Brian A. Van Tine

SUPPLEMENTAL INFORMATION

Supplemental Table 1 – Patient characteristics of osteosarcoma TMA (Related to Figure 1)

**Patient Characteristics**	
**Factor**	**n (%)**
Age at diagnosis, median (range)	18 (4-90)
Gender	
Male	153 (59)
Female	107 (41)
Race	
Asian	9 (3)
Black	35 (14)
Hispanic	75 (29)
White	140 (54)
Stage at presentation	
Localized	215
Metastatic	45
Primary Site	
Femur	140 (54)
Tibia	43 (17)
Fibula	10 (4)
Humerus	32 (12)
Radius/ulna	3 (1)
Mandible	1 (0)
Rib/chest wall	7 (3)
Pelvis/acetabulum	18 (7)
Other	5 (2)
Histologic Subtype	
Osteoblastic	110 (42)
Chondroblastic	49 (19)
Fibroblastic	46 (18)
Telangiectatic	23 (9)
Dedifferentiated parosteal	12 (5)
Small cell	6 (2)
High grade surface	3 (1)
Other – high grade	6 (2)
Other – intermediate/low grade	5 (2)
Radiation Induced Osteosarcoma	
No	250 (97)
Yes	8 (3)
Grade	
Low	6 (2)
Intermediate	1 (0)
High	253 (97)
Pathologic Response (patients receiving pre-op chemo)	
Good (≥ 90 % necrosis)	108 (45)
Poor (< 90 % necrosis)	133 (55)

Supplemental Table 2 – Metabolomics Data Set (separate file) (Related to Figure 2)

Supplemental Table 3 – Lipidomics Data Set (separate file) (Related to Figure 3)

Supplemental Table 4 – NanoString Metabolism Gene Panel (separate file) (Related to Figure 4)

Supplemental Figure 1 – Osteosarcomas demonstrate correlation between PHGDH RNA expression and poor survival (Related to Figure 1)

A

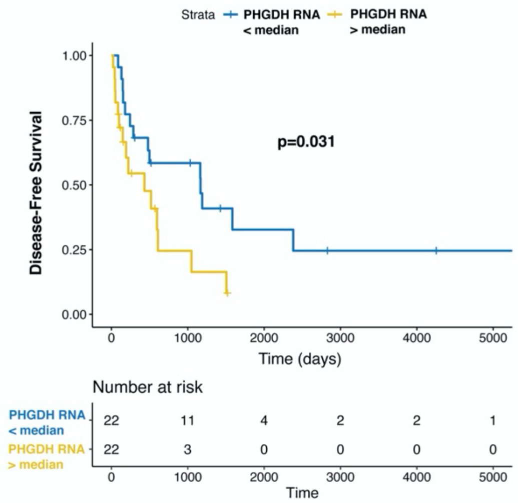

C

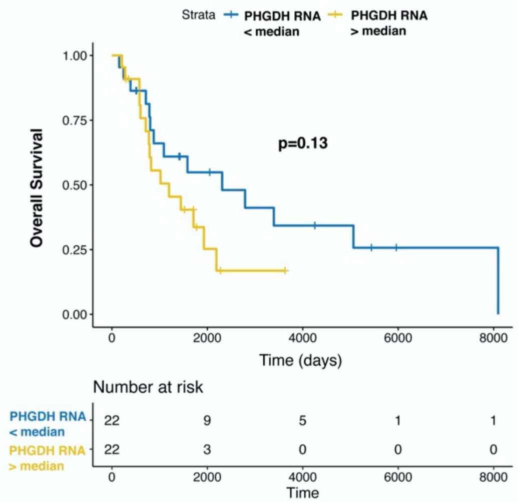

B

Patient Characteristics	
**Factor**	**n (%)**
Age at diagnosis, median (range)	27 (5-81)
Gender	
Male	27 (64)
Female	15 (36)
Race	
Asian	2 (5)
Black	4 (10)
Hispanic	9 (21)
White	27 (64)
Histologic Subtype	
Osteoblastic	11 (26)
Chondroblastic	6 (14)
Fibroblastic	8 (19)
Telangiectatic	2 (5)
Dedifferentiated	5 (10)
Extraosseous	6 (14)
Surface	1 (2)
Giant Cell Rich	1 (2)
High Grade NOS	3 (7)
Radiation Induced Osteosarcoma	
No	39 (93)
Yes	3 (7)
Pathologic Response (patients receiving pre-op chemo)	
Good (90 % necrosis)	10 (24)
Poor (< 90 % necrosis)	32 (76)

Supplemental Figure 1 – Osteosarcomas demonstrate correlation between PHGDH RNA expression and poor survival (Related to Figure 1)

(A) Disease-free survival for osteosarcoma patients with PHGDH RNA levels below the median (blue) compared to PHGDH RNA levels above the median (yellow).

(B) Patient characteristics of osteosarcoma patients corresponding to RNA data.

(C) Overall survival for osteosarcoma patients with PHGDH RNA levels below the median (blue) compared to PHGDH RNA levels above the median (yellow).

Supplemental Figure 2 – PHGDH inhibition causes attenuation of cellular proliferation and TCA cycle activity and increases glycolysis (Related to Figure 2)

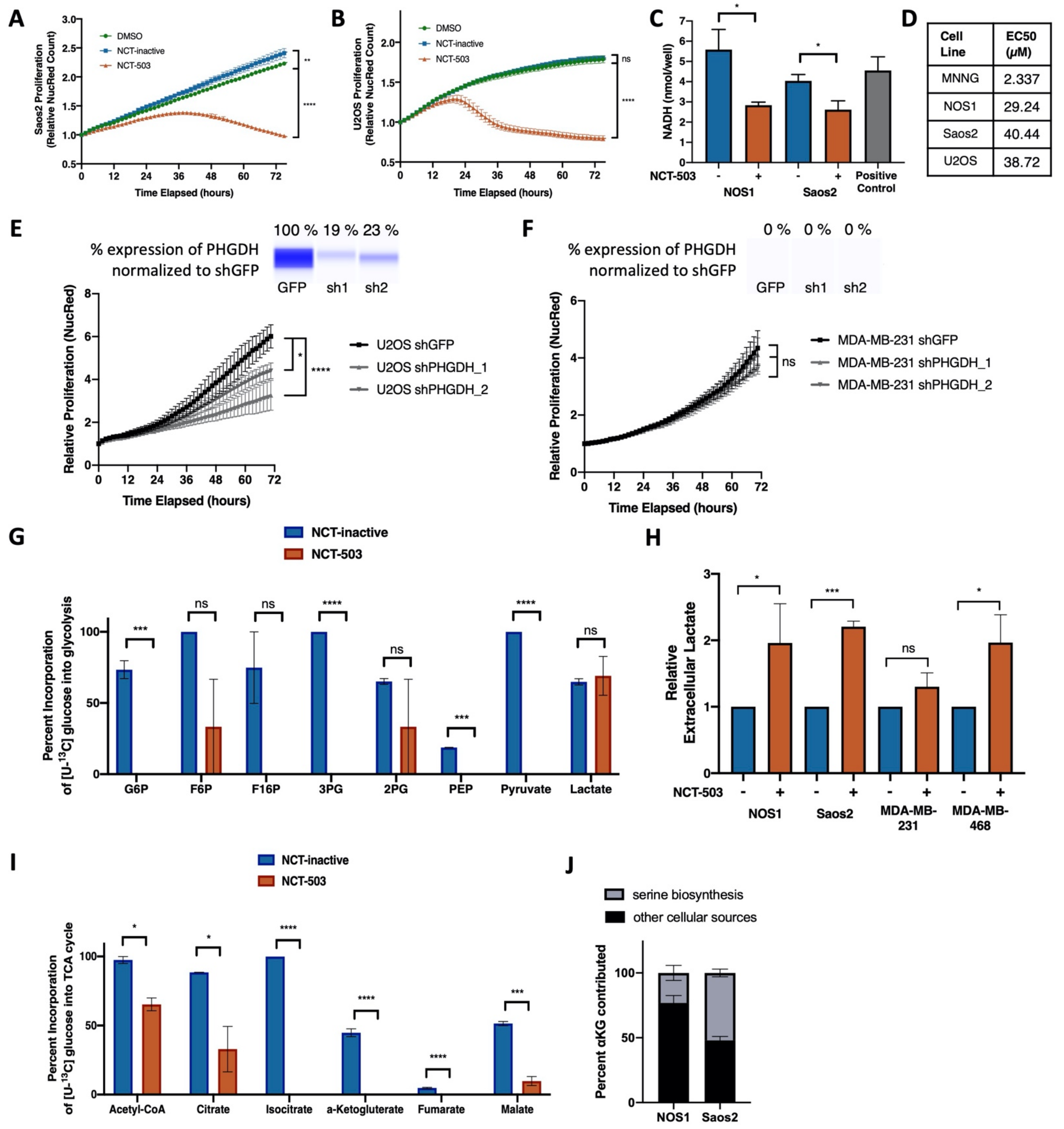

Supplemental Figure 2 – PHGDH inhibition causes attenuation of cellular proliferation and TCA cycle activity and increases glycolysis (Related to Figure 2)

(A) Relative count of red nuclear staining in Saos2 and (B) U2OS cells treated with DMSO (vehicle control), 10 µM NCT-503 inactive control, or 15 µM NCT-503.

(C) NADH produced by PHGDH in presence of 10 µM NCT-inactive, 15 µM NCT-503, or positive assay control.

(D) Calculated EC-50 of NCT-503 for osteosarcoma cell lines.

(E) Relative count of red nuclear staining in U2OS shGFP, shPHGDH_1, and shPHGDH_2 cells and (F) MDA-MB-231 shGFP, shPHGDH_1, and shPHGDH_2 cells. Corresponding protein expression of PHGDH in cell lysates, analyzed using the WES automated capillary blotting system and normalized to total protein levels within the capillary. Band image is representative. Percent PHGDH was calculated using shGFP as 100 %.

(G) Percent incorporation of [U-^{13}C] labeled glucose into glycolytic metabolites in presence of 10 µM NCT-inactive or 15 µM NCT-503, measured by mass spectrometry.

(H) Relative levels of extracellular lactate in presence of 10 µM NCT-inactive or 15 µM NCT-503.

(I) Percent incorporation of [U-^{13}C] labeled glucose into TCA cycle metabolites in presence of 10 µM NCT-inactive or 15 µM NCT-503, measured by mass spectrometry.

(J) Percent of αKG produced in presence of 10 µM NCT-inactive (all cellular sources) or 15 µM NCT-503 (serine synthesis pathway). Percent of αKG produced by cellular sources not including serine synthesis was obtained by subtracting serine-derived αKG from total αKG.

Abbreviations: G6P, glucose-6-phosphate; F6P, fructose-6-phosphate; F16P, fructose-1,6-bisphosphate; 3PG, 3-phosphoglycerate; 2PG, 2-phosphoglycerate; PEP, phosphoenolpyruvate. Bars represent mean of values; error bars represent SEM. All assays were conducted with n = three replicates. Asterisks represent p-values: * < 0.05, ** < 0.01, *** < 0.005, **** < 0.001.

Supplemental Figure 3 – Osteosarcoma cells do not utilize glutamine metabolism at baseline, but accumulate fatty acids in the presence of NCT-503 (Related to Figure 3)

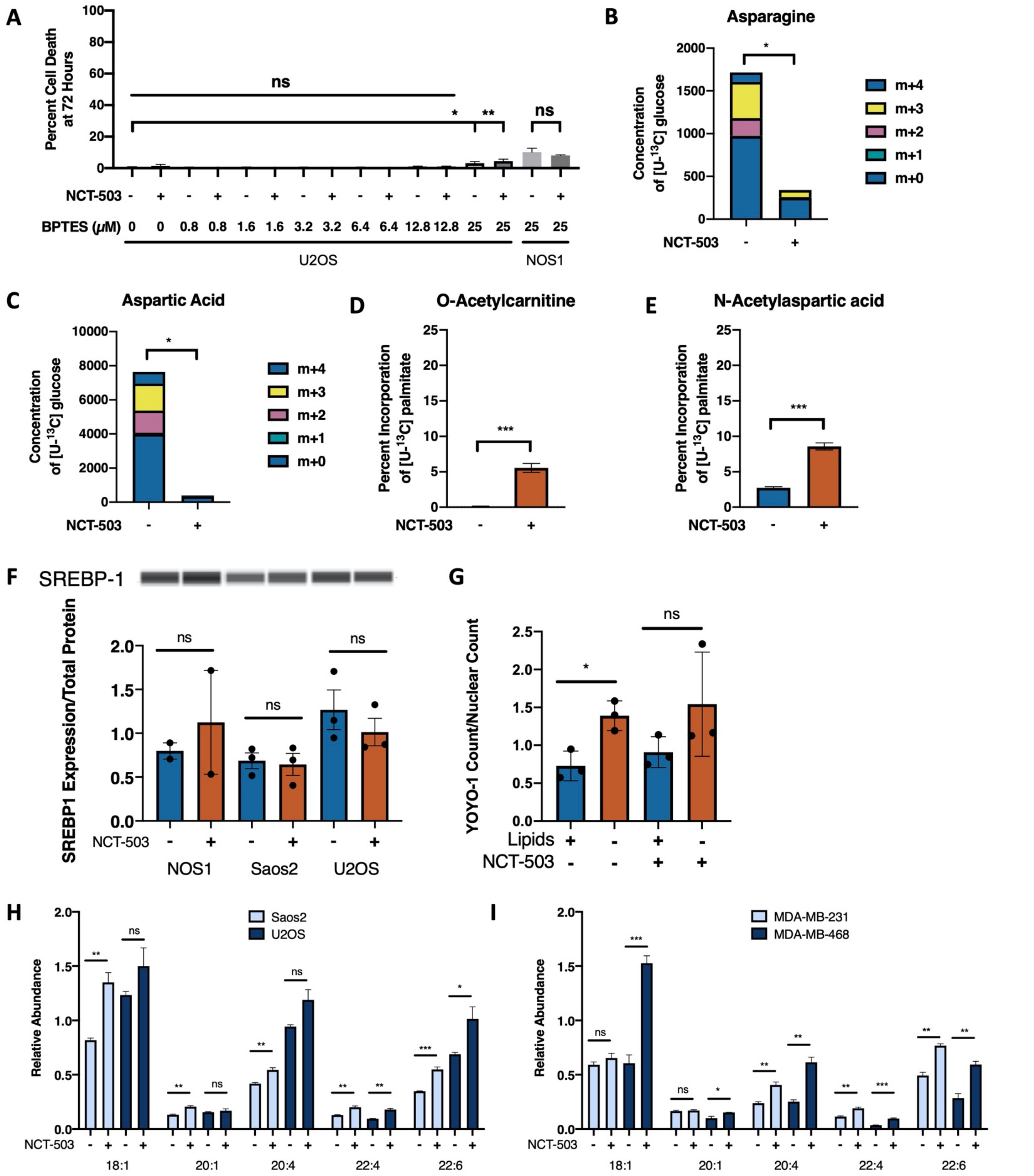

Supplemental Figure 3 – Osteosarcoma cells do not utilize glutamine metabolism at baseline, but accumulate fatty acids in the presence of NCT-503 (Related to Figure 3)

(A) Percent cell death at 72 hours in U2OS cells treated with increasing doses of BPTES and NOS1 cells treated with highest dose of BPTES, with or without 15 μM NCT-503, measured using YOYO-1 Iodide counts/mm^2, normalized to nuclear red count.

(B) Concentration of ^{13}C and unlabeled C in asparagine and (C) aspartate in presence of 10 μM NCT-inactive or 15 μM NCT-503, measured by mass spectrometry.

(D) Percent incorporation of [U-^{13}C] labeled palmitate into O-acetylcarnitine and (E) N-acetylaspartic acid in presence of 10 μM NCT-inactive or 15 μM NCT-503, measured by mass spectrometry.

(F) Protein expression of SREBP-1 in NOS1 (n=2), Saos2 (n=3), and U2OS (n=3) cell lysates treated with either NCT-inactive or NCT-503. Cell lysates were analyzed using the WES automated capillary blotting system and normalized to total protein levels within the capillary. Band image is representative.

(G) Fold change cell death in Saos2 cells cultured with media containing normal FBS or lipoprotein-depleted FBS, and treated with NCT-503 inactive control or NCT-503.

(H) Relative abundance by peak area ratio of five most abundant unsaturated fatty acids in Saos2 and U2OS and (I) MDA-MB-231 and MDA-MB-468.

Bars represent mean of values; error bars represent SEM. All assays were conducted with n = three replicates unless otherwise specified. Asterisks represent p-values: * < 0.05, ** < 0.01, *** < 0.005, **** < 0.001.

Supplemental Figure 4 – NCT-503 modulates amino acid metabolism and transporter gene expression (Related to Figure 4)

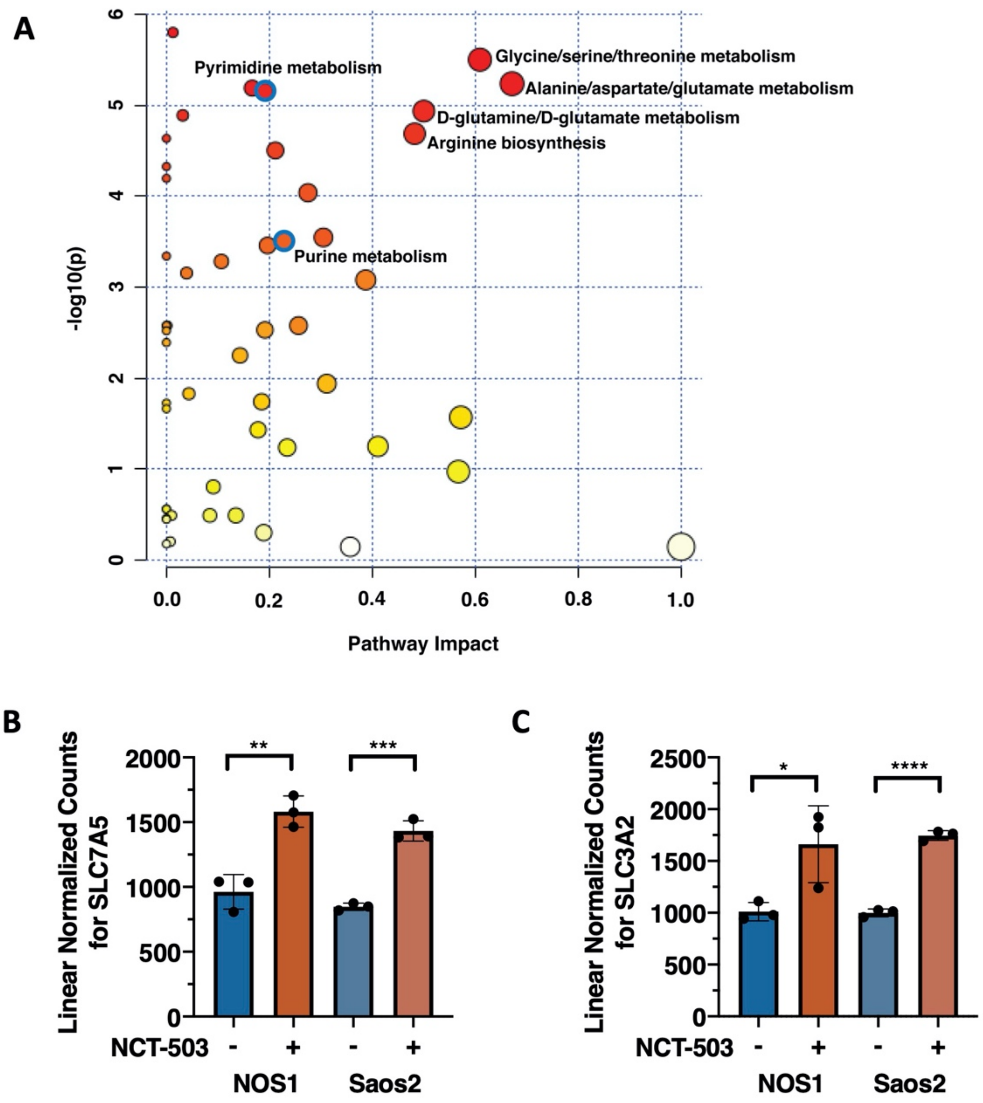

Supplemental Figure 4 – NCT-503 modulates amino acid metabolism and transporter gene expression (Related to Figure 4)

(A) Plot of pathway impact vs. -log10(p value) for amino acid metabolism pathways affected by NCT-503 treatment in NOS1 cells. Analysis conducted using MetaboAnalyst 4.0.

(B) Linear normalized gene counts for SLC7A5 and (C) SLC3A2 in NOS1 and Saos2 cells treated with NCT-inactive or NCT-503. Bars represent mean of values; error bars represent SEM. All assays were conducted with n = three replicates. Asterisks represent p-values: * < 0.05, ** < 0.01, *** < 0.005, **** < 0.001.

Supplemental Figure 5 – Unique biology of perhexiline as a non-rapalog mTORC1 inhibitor causes significant cell death when combined with NCT-503 in PHGDH-high cancer cell lines (Related to Figure 5)

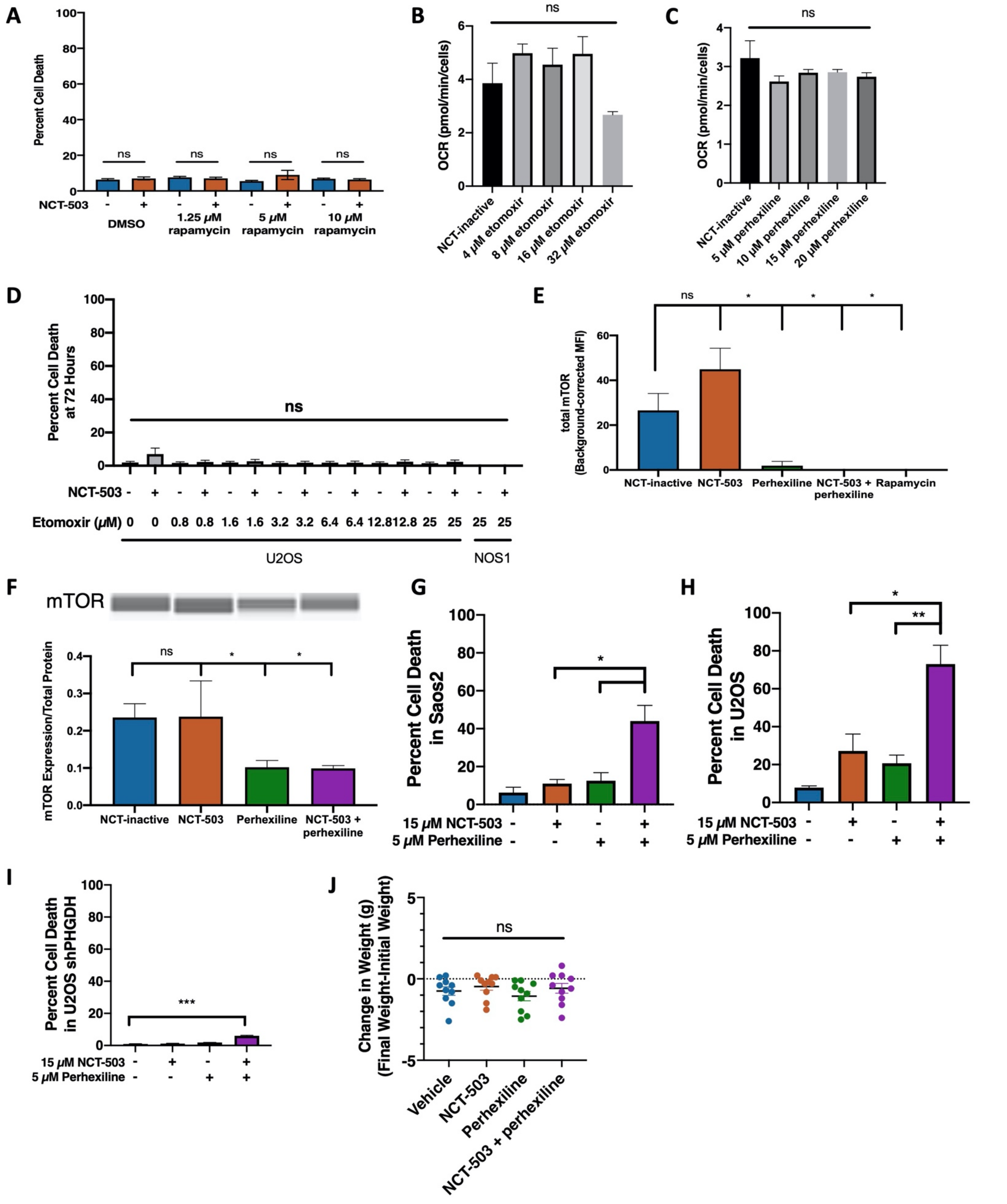

Supplemental Figure 5 – Unique biology of perhexiline as a non-rapalog mTORC1 inhibitor causes significant cell death when combined with NCT-503 in PHGDH-high cancer cell lines (Related to Figure 5)

(A) Percent cell death with increasing doses of rapamycin combined with 15 μM NCT- in NOS1 cells.

(B) Oxygen consumption rate (OCR) for Saos2 cells with increasing doses of etomoxir or (C) perhexiline.

(D) Percent cell death at 72 hours in U2OS cells treated with increasing doses of etomoxir and NOS1 cells treated with highest dose of etomoxir, with or without 15 μM NCT-503, measured using YOYO-1 Iodide counts/mm^2, normalized to nuclear red count.

(E) Total protein levels of mTOR in NOS1 cells treated with NCT-inactive, NCT-503, perhexiline, NCT-503 and perhexiline, or rapamycin.

(F) Protein expression of mTOR in NOS1 cell lysates treated with NCT-inactive, NCT-503, perhexiline, or NCT-503 and perhexiline. Cell lysates were analyzed using the WES automated capillary blotting system and normalized to total protein levels within the capillary. Band image is representative.

(G) Percent cell death at 72 hours in Saos2, (H) U2OS cells, and (I) U2OS shPHGDH cells treated with NCT-inactive, NCT-503, 5 μM perhexiline, or a combination of NCT-503 and 5 μM perhexiline, measured using YOYO-1 Iodide counts/well, normalized to total cell count using red nuclear staining.

(J) Change in mouse weight, calculated as final weight (g) – starting weight (g) for mice treated with vehicle (n = 10), NCT-503 (n = 10), perhexiline (n = 10), or NCT-503 combined with perhexiline (n = 10).

Bars represent mean of values; error bars represent SEM. All assays were conducted with n = three replicates. Asterisks represent p-values: * < 0.05, ** < 0.01, *** < 0.005, **** < 0.001.

Supplemental Figure 6 – NPRL2 and ITFG2 localize to lysosome with perhexiline treatment in PHGDH-negative breast cancer (Related to Figure 6)

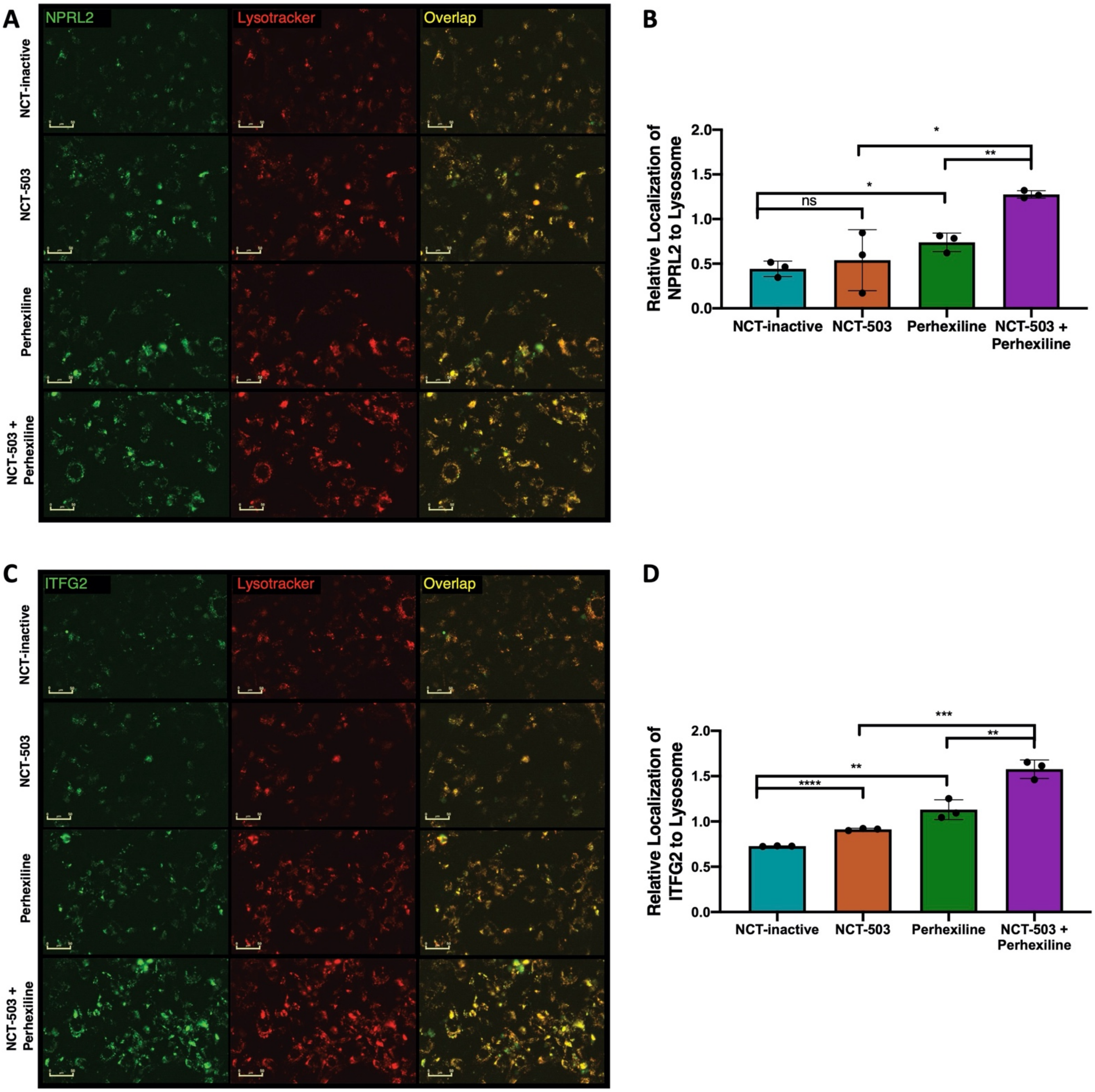

Supplemental Figure 6 – NPRL2 and ITFG2 localize to lysosome with perhexiline treatment in PHGDH-negative breast cancer (Related to Figure 6)

(A) Images of MDA-MB-231 cells, labeled with fluorescent antibody against NPRL2 (GATOR1 component) (green), fluorescent Lysotracker dye (red), and overlay of green and red showing NPRL2 localization at lysosome (yellow) in various conditions: NCT-inactive, NCT-503, perhexiline, and NCT-503 combined with perhexiline for 48 hours. Scale bars represent 50 μm.

(B) Quantification of overlap of NPRL2 antibody with Lysotracker, normalized to cell count.

(C) Images of MDA-MB-231 cells labeled with fluorescent antibody against ITFG2 (KICSTOR component) (green), fluorescent Lysotracker dye (red), and overlay of green and red showing ITFG2 localization at lysosome (yellow) in various conditions: NCT-inactive, NCT-503, perhexiline, and NCT-503 combined with perhexiline for 48 hours. Scale bars represent 50 μm.

(D) Quantification of overlap of ITFG2 antibody with Lysotracker, normalized to cell count.

Bars represent mean of values; error bars represent SEM. All assays were conducted with n = three replicates. Asterisks represent p-values: * < 0.05, ** < 0.01, *** < 0.005, **** < 0.001.
